# Evaluating COVID-19 vaccine allocation policies using Bayesian m-top exploration

**DOI:** 10.1038/s41598-026-40787-x

**Published:** 2026-04-05

**Authors:** Alexandra Cimpean, Timothy Verstraeten, Lander Willem, Niel Hens, Ann Nowé, Pieter Libin

**Affiliations:** 1 Artificial Intelligence Lab, Department of Computer Science, Vrije Universiteit Brussel, Brussels, Belgium; 2 Data Science Institute, Interuniversity Institute of Biostatistics and statistical Bioinformatics, UHasselt, Hasselt, Belgium; 3 Centre for Health Economics Research and Modelling Infectious Diseases, Vaccine & Infectious Disease Institute, University of Antwerp, Antwerp, Belgium; 4 Department of Family Medicine and Population Health (FAMPOP), University of Antwerp, Antwerp, Belgium

**Keywords:** COVID-19, Individual-based models, M-top anytime decision making, Multi-armed bandits, Vaccine policies, Computational biology and bioinformatics, Diseases, Health care, Mathematics and computing

## Abstract

Individual-based epidemiological models support the study of fine-grained preventive measures, such as tailored vaccine allocation policies, in silico. As individual-based models are computationally intensive, it is pivotal to identify optimal strategies within a reasonable computational budget. Moreover, due to the high societal impact associated with the implementation of preventive strategies, uncertainty regarding decisions should be communicated to policy makers, which is naturally embedded in a Bayesian approach. We present a novel technique for evaluating vaccine allocation strategies using a multi-armed bandit framework in combination with a Bayesian anytime *m*-top exploration algorithm. *m*-top exploration allows the algorithm to learn *m* policies for which it expects the highest utility, enabling experts to further inspect this small set of alternative strategies, along with their quantified uncertainty. The anytime component provides policy advisors with flexibility regarding the computation time and desired confidence, which is important as it is difficult to make this trade-off beforehand. We consider the Belgian COVID-19 epidemic using the individual-based model STRIDE, where we learn a set of vaccination policies that minimise infections and hospitalisations. In this setting, each policy specifies how the limited weekly supply of different COVID-19 vaccine types is allocated across age groups over the course of the vaccination campaign, under given social contact reduction policies. Formally, we define each such unique allocation policy as an arm within our multi-armed bandit framework. Through experiments we show that our method efficiently identifies the *m*-top policies. Finally, we explore how vaccination policies can best be organised under different contact reduction schemes and vaccine uptake proportions. We show that the top policies follow a clear trend regarding prioritised age groups and assigned vaccine types, which provides insights for future vaccination campaigns. Furthermore, our experiments suggest that the uptake proportion has only a limited influence on overall policy optimality.

## Introduction

Epidemiological models, such as compartmental and individual-based models (IBMs), are critical tools for evaluating the impact of preventive measures in silico^1,2^. While IBMs typically involve greater complexity and computational cost than compartmental models, they offer more realistic assessments of intervention strategies^3^, provided they are well informed^4^. To leverage these advantages at scale, it is essential to optimise computational efficiency.

Traditionally, the literature evaluates a fixed set of preventive strategies by simulating each one the same number of times^5–7^. However, this uniform allocation of resources is inefficient for identifying optimal strategies, as significant computation is often spent on suboptimal options. Moreover, there is no consensus on how many simulations per strategy are needed^8^, and this number depends on the inherent difficulty of the evaluation task^9^. Given that a single IBM run can take minutes to hours, depending on its complexity, reducing the number of required simulations can drastically lower the total computational burden. This enables the practical use of IBMs in studies that would otherwise be infeasible, and allows existing studies to explore a broader range of scenarios. Broadening the scope of analysis is especially valuable, as it increases confidence in the robustness and generalisability of recommended preventive strategies^10^.

We present a novel technique for evaluating vaccine allocation strategies using a multi-armed bandit framework in combination with a Bayesian anytime *m*-top exploration algorithm. Here, *m* denotes a pre-specified integer representing the number of top-performing strategies the policymaker wishes to identify (e.g., the top-5 or top-10 best options). Unlike traditional optimisation that seeks a single best solution, *m*-top exploration allows the algorithm to learn a set of *m* policies for which it expects the highest utility. This enables experts to inspect this small, high-quality set of alternative strategies, along with their quantified uncertainty. The anytime component provides the policy advisors with flexibility regarding the time at which a decision is made. This is especially important when computationally intensive models are used as for such models it is difficult to make a trade-off between the available budget and desired confidence. We focus on a Bayesian learning approach, to quantify the uncertainty of the decision making.

**Fig. 1:**
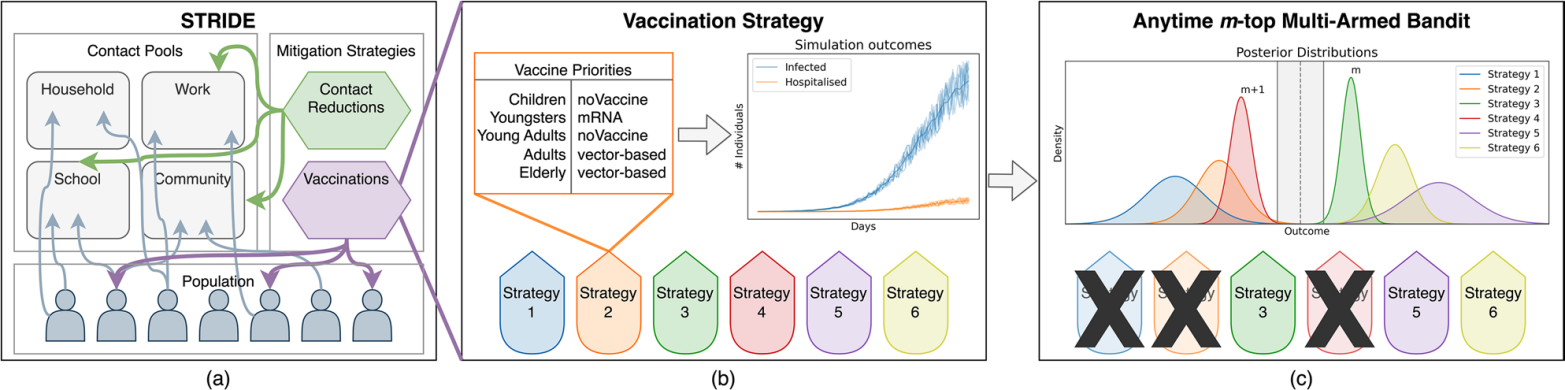
Visual representation of the research approach. In (**a**), we show a high-level overview of the STRIDE individual-based model, where each individual in the population participates in certain contact pools. These contact pools (i.e., Household, Work, School, Community) represent the specific social environments where individuals interact and transmission events are simulated. The epidemic mitigation measures utilised are contact reductions and vaccines. In (**b**), we compare different vaccination strategies, each with its own prioritisation of vaccine types across age groups, where noVaccine signifies that the age group is not prioritised. Each unique vaccine allocation strategy shown here corresponds to a single arm in the bandit process. Using STRIDE, we can simulate each vaccine strategy. We apply a Bayesian anytime *m*-top multi-armed bandit algorithm, as shown in (**c**), to efficiently explore the intervention strategies to identify the top *m* strategies, by focussing on the decision boundary shown in grey. As the bandit explores strategies close to the decision boundary, it reduces its uncertainty about the top *m* strategies with the highest estimated utility, to the right of this boundary.

Using this innovative framework, we study a vaccine allocation problem, where we investigate how the weekly supply of COVID-19 vaccines in Belgium could have been optimally allocated to the different age groups in the population. As vaccines are administered gradually, certain contact reductions remained in place during the vaccination campaign to curb the disease burden. Whether the design of social contact restrictions affects the optimal vaccine allocation, is part of our experimental exploration. Moreover, we investigate the impact of the vaccine uptake proportion (i.e., the proportion of individuals that will comply with the strategy and take the vaccine) on the design of vaccine allocation strategies^11^. In this regard, we study the impact of household clustering of unvaccinated individuals^12^. To evaluate detailed contact reduction schemes and vaccine uptake on a household level, we adopted the fine-grained open-source individual-based model STRIDE, which has previously been used in COVID-19 modeling analyses^12–15^. In this framework, transmission dynamics are driven by contact pools, i.e., distinct social environments including Households, Schools, Workplaces, and the Community. These pools define the specific settings where individuals interact and infection events occur, governed by age-stratified contact rates^13^. Formally, we define each such unique vaccine allocation strategy as an arm within our multi-armed bandit framework. Figure 1 visualises our research approach, including the STRIDE individual-based model (panel (a)), the vaccine allocation strategies (panel (b)), and the anytime *m*-top Bayesian multi-armed bandit (panel (c)).

While age-stratified vaccine allocation has been studied using compartmental models, this work introduces four distinct contributions that bridge the gap between computational efficiency and realistic epidemiological modelling.

1. Explicit social contact patterns: By adopting an IBM that explicitly accounts for social contact patterns within households, schools, workplaces, and the general community, we capture the local clustering of transmission events. While age-specific mixing is commonly studied in compartmental models, the topology of the social network also plays a crucial role. This relates to the dynamic in which households serve as bridges for transmission between schools and workplaces. Additionally, we assume that vaccine sentiments are clustered and consequently model uptake at the household level.
2. Joint analysis of non-pharmaceutical interventions (NPIs), uptake, and allocation: Unlike studies that optimise allocation in isolation, we analyse the joint interaction between vaccine allocation, a diverse set of dynamic social contact reductions (non-pharmaceutical interventions; NPIs), and household-level vaccine uptake. Our results show that the optimal allocation is not static but conditional on the specific NPI regime in place (e.g., contact reductions in schools vs. workplaces). This level of conditional insight is difficult to capture given the population-averaged mixing inherent to compartmental models (even when age-stratified), highlighting the benefits of using a model with a higher granularity such as an individual-based model.
3. Anytime bandits for stochastic IBMs: Unlike compartmental models, high-fidelity IBMs capture fine-grained transmission dynamics but are typically too computationally expensive for traditional optimisation. While previous work looked into fixed-budget best-arm identification methods^9^, this is the first work to consider an anytime bandit framework to learn optimal mitigation strategies. This allows policymakers to stop the learning process at any point to inspect the current top strategies, offering critical flexibility between computational budget and decision confidence.
4. Policy-centric uncertainty quantification: Standard optimisation seeks a single global maximum, which can be brittle to model assumptions. By formulating the problem as *m*-top exploration, we identify a set of high-performing strategies rather than a single best option. This provides policymakers with a robust portfolio of alternatives, accompanied by quantified uncertainty, facilitating decision-making that accounts for logistical and political constraints. Furthermore, by formulating the problem in a Bayesian framework, we leverage epidemiological priors to improve sample efficiency. The resulting posterior distributions provide policymakers with a transparent view of the decision uncertainty.

## Related work

Epidemic control has been explored in a reinforcement learning setting, both from a stateful and a multi-armed bandit perspective. From a stateful reinforcement learning perspective, the concept of learning dynamic policies by formulating the decision problem as a Markov decision process (MDP) was first introduced by Yaesoubi and Cohen^16^. To investigate dynamic tuberculosis case-finding policies in HIV/tuberculosis co-epidemics, a policy iteration algorithm was used to solve the MDP^17^. This technique was later extended to include cost-effectiveness in the analysis and applied to mitigation policies (that is, school closures and vaccines) in the context of pandemic influenza in a simplified epidemiological model^18^. More recently, Libin et al. used deep reinforcement learning to learn mitigation strategies in the context of pandemic influenza^19^. Reymond et al. explored COVID-19 mitigation policies from a multi-objective reinforcement learning perspective, where complex mitigation policies with possibly conflicting objectives are balanced to learn the best trade-offs^20^.

From a multi-armed bandit perspective, we distinguish efforts that investigate a cumulative regret and a best-arm identification setting. On the one hand, in the cumulative regret setting, we identified work focusing on various preventive strategies in the context of COVID-19^21–23^. We note that these studies do not consider individual-based models. On the other hand, best-arm identification algorithms have been used to evaluate preventive strategies in individual-based models^9^, which we consider the work most closely related to our study. In that work, Bayesian fixed-budget best-arm identification algorithms are used to evaluate preventive strategies in the context of pandemic influenza. A broad overview on the state of the art with respect to (Bayesian) best-arm identification algorithms is provided by Kaufmann et al. and Hoffman et al.^24,25^.

However, the use of fixed-budget best-arm identification has some important limitations. First, simply returning the single best prevention strategy can be an obstacle for public health scientists, as this implies that public health scientists can only offer a take-it-or-leave-it option to government officials, rather than a set of options that can be evaluated within the political and legal framework of the government. Additionally, from a health economics perspective, a set of optimal policies can be used to negotiate a fair cost with the producers of pharmaceutical supplies. Second, Libin et al. assume a fixed computational budget, that needs to be specified a priori^9^. We argue that deciding the budget upfront can be challenging, which is especially the case when computationally expensive models are used, for which it is difficult to make a trade-off between the available budget and desired confidence. As such, we assert that an anytime bandit setting can overcome these limitations, as an initial budget can still be provided, but the budget can be extended when necessary. To address these limitation, in this work, we study the anytime *m*-top exploration problem, introduced by Jun et al.^26^. Jun et al. introduce the frequentist algorithm AT-LUCB^26^. We note that as a UCB-variant, AT-LUCB is not equipped to incorporate prior knowledge with respect to the reward distribution. As Libin et al. have shown that incorporating such knowledge can greatly improve the learning performance^9^, we study a Thompson sampling algorithm to solve the anytime *m*-top exploration problem: Boundary Focused Thompson sampling^27^.

## Methods

### Epidemic bandits

We formulate the evaluation of preventive strategies as a multi-armed bandit problem^9^, with the aim of identifying the *m*-top arms using anytime decision making algorithms^27^. The presented method is generic, capable of dealing with different epidemic model types, that consider distinct pathogens, contact networks and preventive strategies. This method will be evaluated in the context of COVID-19 in the next section.

First, we formally define the multi-armed bandit.

#### Definition 1

(Multi-armed bandit) A *multi-armed bandit* involves *K* arms that can be pulled^28^, where each arm $a_k$ has a *reward distribution*. When an arm $a_k$ is pulled, it returns a reward $r_k$ sampled from $a_k$’s reward distribution. For each arm $a_k$ we have the expected reward $\mu _k = \mathop {\mathrm {\mathbb {E}}}\limits \left[ r_k\right]$.

A common use of the multi-armed bandit is to pull a sequence of arms such that the best arm is identified. However, in this work, our aim is to solve the *m*-top exploration problem ($m < K$), where the objective is to identify the *m* best arms, with respect to the expected reward $\mu _k$ of the arms^29^. Formally, we have $\mu _1 \ge \ldots \ge \mu _m \ge \mu _{m+1} \ge \ldots \ge \mu _K$, and the objective is to identify the set $\{\mu _1, \ldots , \mu _m \}$. This is a *pure exploration* problem where the focus is on gaining knowledge about which *m* arms are ranked the highest. Next, we provide a formal definition of the epidemic model we consider^9^.

#### Definition 2

(Stochastic epidemiological model) A *stochastic epidemiological model* $\mathscr {E}$ is defined in terms of a model configuration $c \in \mathscr {C}$ and can be used to evaluate a preventive strategy *p*. The result of a model evaluation is referred to as the *model outcome*. Evaluating the model $\mathscr {E}$ thus results in a sample of the model’s *outcome distribution*:

1 $$\begin{aligned} \text {outcome} \sim \mathscr {E}(c, p) \end{aligned}$$

The model outcome can be any statistic relevant to the decision maker, such as prevalence, proportion of symptomatic individuals, proportion of hospitalised individuals, mortality or societal cost. Note that a model configuration $c \in \mathscr {C}$ describes the complete model environment, i.e., both aspects inherent to the model and options that the modeller can provide (e.g., population statistics, vaccine properties).

Our objective is to find the set of *m*-top preventive strategies (i.e., the strategies that minimise the expected outcome) from a set of alternative strategies

2 $$\begin{aligned} \{p_1,...,p_K\}, \end{aligned}$$

for a particular configuration

3 $$\begin{aligned} c_0 \in \mathscr {C}, \end{aligned}$$

where $c_0$ corresponds to the context of the studied epidemic. To this end, we consider a multi-armed bandit with preventive strategies $\{p_1,...,p_{K}\}$ represented by arms $\{a_1,...,a_{K}\}$. Pulling arm $a_k$ corresponds to evaluating the corresponding preventive strategy $p_k$, by running a simulation in the epidemiological model $\mathscr {E}(c_0, p_k)$. The bandit thus has preventive strategies as arms with reward distributions corresponding to the outcome distribution of an epidemiological model $\mathscr {E}(c_0, p_k)$. To make this concrete, the preventive strategy $p_k$ represents a specific epidemiological intervention. For example, in a mitigation study, an arm $p_k$ could represent a specific closure threshold (e.g., close schools when daily incidence exceeds 50). In a testing study, an arm might represent a testing frequency (e.g., test healthcare workers every 3 days). In the COVID-19 vaccine allocation study presented later in this paper, $p_k$ represents a specific prioritisation logic, defining which vaccine types are assigned to which age groups. While the parameters of the outcome distribution (i.e., the parameters of the epidemiological model) are known, it is intractable to determine the top strategies analytically. Hence, we must learn about the outcome distribution via interaction with the epidemiological model.

### *m*-Top exploration

Our objective is to identify the *m*-top preventive strategies for a particular configuration of an epidemiological model. We consider two anytime *m*-top algorithms: AnyTime Lower and Upper Confidence Bound (AT-LUCB) and Boundary Focused Thompson Sampling (BFTS).

#### AnyTime lower and upper confidence bound algorithm

The AT-LUCB algorithm invokes the fixed-confidence LUCB algorithm^26,30^. At each time step *t*, AT-LUCB (Algorithm 1) returns the empirical *m*-top arms $J^{(t)}$. Given *K* number of arms, $\hat{\mu }_a^{(t)}$ is the empirical mean for arm *a* at time step *t*. The amount of times arm *a* was pulled until time *t* is denoted by $n_a^{(t)}$. Given the LUCB stage index *s*, the confidence parameter at stage *s* is determined by a decaying failure parameter $\delta _s=\delta _1\alpha ^{(s-1)}$. The stage to which time *t* belongs is defined as $S^{(t)}$.

**Algorithm 1:**
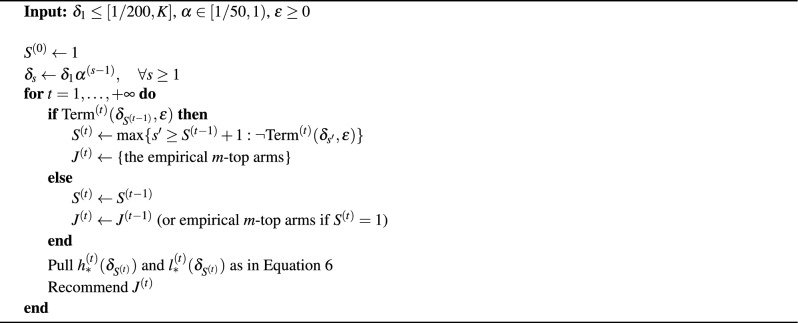
AT-LUCB.

The exploration strategy of AT-LUCB relies on the upper confidence bound $U_a^{(t)}$ and lower confidence bound $L_a^{(t)}$:

4 $$\begin{aligned} \begin{aligned}&U_a^{(t)}(\delta _s) = \hat{\mu }_a^{(t)} + \beta (n_a^{(t)},t,\delta _s)\\&L_a^{(t)}(\delta _s) = \hat{\mu }_a^{(t)} - \beta (n_a^{(t)},t,\delta _s), \end{aligned} \end{aligned}$$

with,

5 $$\begin{aligned} \beta (n_a^{(t)},t,\delta _s)=\sqrt{\frac{1}{2n_a^{(t)}}\ln \left( \frac{5}{4}\frac{K \cdot t^4}{\delta _s}\right) }. \end{aligned}$$

Each time step *t*, the algorithm pulls arms

6 $$\begin{aligned} \begin{aligned}&h_*^{(t)}(\delta _{S^{(t)}}) = \arg \min _{a \in \textrm{High}^{(t)}}L^{(t)}_a(\delta )\\&l_*^{(t)}(\delta _{S^{(t)}}) = \arg \max _{a \in \textrm{High}^{(t)}}U^{(t)}_a(\delta ), \end{aligned} \end{aligned}$$

with $\textrm{High}^{(t)}$ the *m*-top arms at time $t - 1$. When the terminating condition $\textrm{Term}^{(t)}(\delta , \epsilon ) = \{ U^{(t)}_{l_*^{(t)}(\delta )}(\delta ) - L^{(t)}_{h_*^{(t)}(\delta )}(\delta ) < \epsilon \}$ is met, the algorithm moves to the next stage.

#### Boundary focused Thompson sampling

While confidence bound algorithms such as AT-LUCB permit specifying tight theoretical bounds, algorithms based on Thompson sampling typically perform better in practice^31^. Thompson sampling uses samples of the bandit’s posteriors to decide which arm to pull next.

By using a Bayesian *m*-top identification algorithm, prior knowledge about the outcome distributions can be taken into account when defining an appropriate prior and posterior on the arms’ reward distributions. This prior knowledge can increase the sample efficiency while the resulting posteriors provide valuable information about the decision uncertainty to guide policy makers.

For a multi-armed bandit, our prior belief over the arms’ means is given by a prior distribution $\pi (.)$. Given an observed history $\mathscr {H}^{(t-1)}$ of rewards *r* from pulling arms *a* until timestep $t - 1$, where

$$\begin{aligned} \mathscr {H}^{(t-1)} = \left\{ a^{(i)}, r^{(i)}\right\} _{i=1}^{(t-1)}, \end{aligned}$$

the posterior over the means of the bandit is defined as:

$$\begin{aligned} \pi \left( \cdot \ |\ \mathscr {H}^{(t-1)} \right) , \end{aligned}$$

where $\pi (\cdot )$ is conditioned on the observed history.

At each timestep *t*, Thompson sampling samples an estimate $\tilde{\mu }^{(t)}_k$ of the mean $\mu _k$ from each posterior *k* and ranks these samples to select the arm with the highest sampled mean. By sampling from the posterior, Thompson sampling uses the uncertainty of the mean to balance exploration and exploitation. As it is playing an arm multiple times, the posterior’s uncertainty decreases and Thompson sampling will gear towards the highest ranking arms. As Thompson sampling is able to use prior knowledge, sampling efficiency can be greatly improved. In the context of epidemic decision making, we can derive such knowledge using epidemiological modelling theory, which we will do for the experimental scenario considered in Section.

**Fig. 2:**
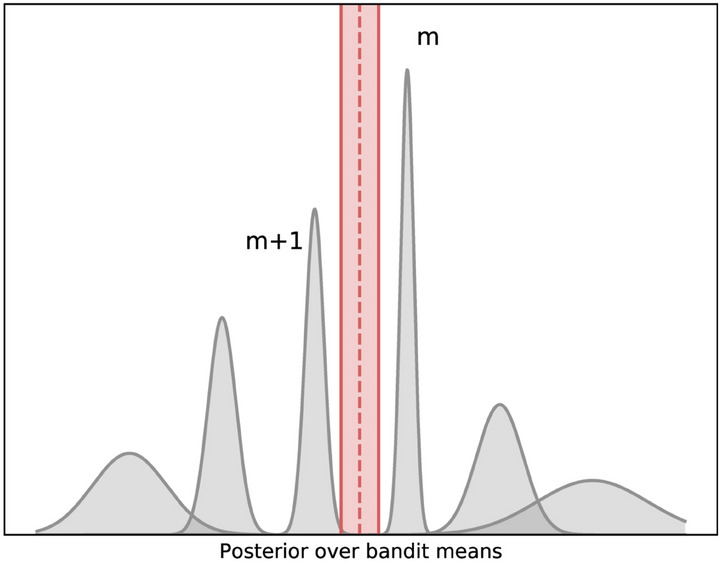
Posteriors for an artificial bandit ($K = 6$, $m = 3$) (gray) and BFTS’ decision boundary (red) with confidence bounds to demonstrate its uncertainty.

Boundary Focused Thompson Sampling (BFTS)^27^ implements a Thompson sampling variant for *m*-top exploration. It uses the posterior samples as an estimate for the arms’ means, which are ranked as in Thompson sampling. BFTS strives to recommend the *m*-top best arms at any given time. To denote the rank of the $\rho$-ordered arm, we define this operator:

7 $$\begin{aligned} \psi _\rho (\tilde{\boldsymbol {\mu }}^{(t)}). \end{aligned}$$

BFTS (Algorithm 2) focuses on both sides of the decision boundary for the *m*-top arms, in order to decrease the uncertainty about arms $a_m^{(t)}$ and $a_{m+1}^{(t)}$ with rankings $\psi _{m}(\tilde{\boldsymbol {\mu }}^{(t)})$ and $\psi _{m+1}(\tilde{\boldsymbol {\mu }}^{(t)})$, respectively. Therefore, the arms ranked $\psi _{m}(\tilde{\boldsymbol {\mu }}^{(t)})$ and $\psi _{m+1}(\tilde{\boldsymbol {\mu }}^{(t)})$ are played with equal probability using a Bernoulli experiment.

A key insight regarding BFTS is that its exploration is guided by sampling from the posterior distribution, balancing between $\psi _{m}(\tilde{\boldsymbol {\mu }}^{(t)})$ and $\psi _{m+1}(\tilde{\boldsymbol {\mu }}^{(t)})$, which represent our belief about the decision boundary at time *t*. Since the posterior captures the uncertainty inherent in the bandit problem, sampling from the $m^{\textrm{th}}$ or ${m+1}^{\textrm{th}}$ ordered arm initially promotes exploration across all arms when an uninformative prior is used. Over time, as the uncertainty for the outermost arms decreases, BFTS shifts its focus toward the arms closer to the decision boundary. Figure 2 illustrates this progression in a simple bandit scenario ($K=6$ and $m=3$) using Gaussian posteriors. In Appendix B we provide a Bayesian analysis of BFTS. While this analysis does not result in a bound on the simple regret, it does provide additional insight in BFTS’ exploration strategy and confirms that this strategy is well-grounded.

**Algorithm 2:**
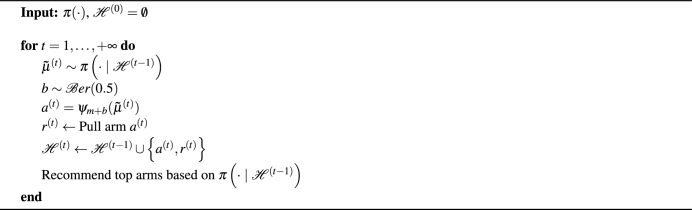
Boundary focused Thompson sampling.

## Vaccine policy evaluation

SARS-CoV-2 has highlighted the importance of pandemic mitigation strategies^32^. This virus manifests in distinct clinical outcomes, ranging from asymptomatic infection to COVID-19 disease, which may induce mild to severe symptoms^32,33^. Severe COVID-19 cases require hospitalisation and might result in a fatal outcome^32,34^. Up to November 2024, over 776 million confirmed cases and 7 million deaths were reported^35^. Since the end of 2019, new variants of SARS-CoV-2 have emerged. The first major mutation, D614G, induced an increased transmissibility and infectiousness, making it the dominant strain of the virus globally^36^. Subsequently, a series of Variants of Concern emerged which further increased transmissibility and/or disease severity^37^. To avoid the overflow of hospitals and to reduce mortality, measures to reduce the number of infections were taken. In the first phase of the pandemic, such interventions were limited to imposing contact reductions^38^. In a later phase of the pandemic, i.e., begin 2021, vaccines became available in many countries^13,32,39^.

In this work, we focus on learning optimal policies to allocate vaccines to a large population when vaccines become available in limited batches, due to the gradual production of vaccines. As vaccines are administered gradually, certain contact reductions need to be kept in place during the vaccination campaign to maintain the disease burden. However, the design of these social contact restrictions, including their focus and intensity, can vary while still maintaining comparable levels of disease burden. Therefore, we evaluate vaccine allocation strategies under different contact reduction scenarios in our experiments. We investigate how to organise COVID-19 vaccine allocation policies targeted at the minimisation of two distinct criteria: infections and hospitalisations. We explore different determinants regarding vaccine allocation policies, including the targeted age group and the vaccine type (i.e., mRNA and vector-based), which results in a large number of preventive policies that is to be evaluated.

We consider the Belgian COVID-19 epidemic in early 2021, where vaccine supplies started to be delivered on a weekly basis, with a changing supply rate as vaccine production increased over time. We take into account the two types of vaccines that were available in Belgium during this phase, i.e., mRNA^40,41^ and vector-based vaccines^42^. We investigate how a weekly supply of vaccines is best allocated among all age groups of the population. We consider different social distancing schemes, under distinct vaccine uptake proportions (i.e., the proportion of individuals that will comply with the strategy and take the vaccine), to explore the effect of such policies on the vaccination campaign. Specifically, we study the impact of household clustering in vaccine uptake^12^. Household clustering is important in this regard, as recent work shows that households constitute a reasonable proxy for predictors associated with vaccine hesitancy^43^. Moreover, parents who have a negative attitude towards vaccination might be reluctant to vaccinate their children^12^.

Children were excluded from the initial COVID-19 vaccination campaigns in 2021 for regulatory reasons. However, they are considered vaccine eligible in our study to enable a population-wide assessment to shape future vaccine allocation strategies, consistent with earlier research^44,45^.

To support detailed contact reduction schemes and investigate vaccine uptake at the household level, the use of a fine-grained individual-based model is warranted^12,15,46^. To this end, we use the STRIDE individual-based simulator^46^, to explicitly model 11 million Belgians^13^, that can engage in social contacts at home, in workplaces, in schools or in the general community. To enable a Bayesian learning approach, we will introduce priors for these scenarios using insights from epidemic modelling theory.

### STRIDE model and configuration

In our experiments, we start the simulation period on January 1st 2021, when the first COVID-19 vaccines became available and the circulating variant in Belgium was the Alpha VoC. We use the individual-based model STRIDE to simulate the entire Belgian population of 11 million individuals. A single simulation considers 4 calendar months, from January 1st 2021 until May 1st 2021, and includes school holidays (January 1st to January 3rd, February 15th to February 21st and April 5th to April 18th). Any chosen vaccination strategy is fixed throughout the simulation, resulting in an aggregate reward at the end of the simulation. Depending on the social contact scenario, a distinct regimen of social contact reductions is imposed on the population. Imposing a higher contact reduction means individuals can participate in fewer person-to-person contacts, thereby reducing their likelihood to acquire infection. We consider different social contact scenarios, as specified in Table 1, to explore whether the vaccination strategy is affected by imposed contact reductions. The model explicitly accounts for contact tracing that was in place and additional details on this can be found in Appendix C.

Social interactions in STRIDE are governed by age-stratified contact rates which define the average number of contacts an individual makes in specific pools (i.e., household, work, school, community) on a given day. These rates are explicitly defined for distinct calendar types, distinguishing between weekdays, weekends, national holidays, and school holiday periods. As contacts in schools are defined based on age in the STRIDE model, we consider primary school to be ages 6-11, secondary schools to be ages 12–17, and tertiary school to be ages 18–25^13^. The contact reductions specified in Table 1 are operationalised as a proportional decrease in the contact probabilities for these specific pools and age groups. Specifically, when an $x\%$ reduction is applied to a setting, the contact rate parameter for that respective pool is scaled by a factor of $(1 - x/100)$. For example, when contacts in secondary schools are reduced by 50%, the contact rate parameter for that respective pool and age group are halved. This means that every student present in the model continues to attend the school pool, but their probability of establishing a social contact relevant for transmission with any other student is halved. This reduces the average number of daily contacts for each individual in that setting, acting as a proxy for the aggregate effect of capacity limits (e.g., hybrid learning) on transmission potential, consistent with the established calibration by Willem et al.^13^.

The contact reduction values in Table 1 were selected to align with specific governmental policies enforced in Belgium during the studied period (early 2021), as well as to explore relevant hypothetical relaxation scenarios grounded in prior modelling work. Primary schools are assumed to be fully open (i.e., 0% contact reduction) across all scenarios, consistent with the policy to prioritise on-site learning for young children^38^. Secondary schools are modelled at 50% reduction in the baseline, reflecting a hybrid system where secondary schools operated at half capacity to limit physical presence, with masks and/or improved ventilation^47^. In the model, this is operationalised as a 50% reduction in contact probability to capture the aggregate decrease in interaction density, as described in Willem et al.^13^. Furthermore, we explore a full reopening (0% contact reduction) to assess the impact of school-based transmission when no precautious measures to prevent transmission were in place. For the Tertiary schools we assume a baseline of 100% closure. The 100% reduction reflects full closure (i.e., full distance learning), which was the policy in place during certain phases of the pandemic^14^. We also explore a scenario with 70% contact reduction, that mimics the ’Code Orange’ policy, where higher education institutions were required to limit physical presence to a maximum capacity^48^. In the model, this is operationalised as a 70% reduction in contact probability to capture the aggregate decrease in interaction density. For the Workplace and Community contact reductions, we used 50% and 70%, in line with earlier work^14^. The aim for the Workplace and Community contact reductions is to explore and compare two distinct, yet substantial, levels of contact reduction.

We use the STRIDE model configuration as calibrated on the Belgian COVID-19 epidemic in earlier work^13^, where the first wave of the COVID-19 pandemic and the exit strategies were studied. Because we simulate the progress of the pandemic starting from January 1st 2021 and not from the start of the pandemic, the population is initialised with the proportion of immunity that was estimated at that moment in Belgium. This proportion of immunity was estimated using the stochastic compartment model by Willem et al.^49^. We consider the Alpha VoC variant of SARS-CoV-2, which is 50% more infectious compared to previously circulating variants^50^.

**Table 1:** Social contact reduction schemes for the epidemic COVID-19 scenarios for Belgium. 0% implies there is no reduction in contacts and 100% means imposing full contact reduction.

Scheme	Primary school	Secondary school	Tertiary school	Workplace	Community
*Baseline*	0%	50%	100%	70%	70%
*Relaxed*	0%	50%	100%	50%	50%
*Tertiary Education*	0%	50%	70%	70%	70%
*Secondary Schools*	0%	0%	100%	70%	70%
*Relaxed Community*	0%	50%	100%	70%	50%
*Relaxed Workplace*	0%	50%	100%	50%	70%

### Vaccine allocation

**Fig. 3:**
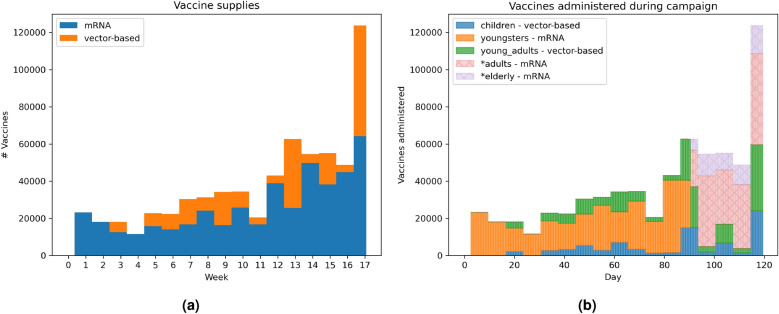
(**a**) Stacked bar chart of the reported vaccine supply from January 1st 2021^53^. (**b**) Stacked bar chart for one example of an uptake strategy where the entire populations accepts vaccines, starting with vaccinating children and young adults with vector-based vaccines and youngsters with mRNA vaccines. When the youngsters are fully vaccinated, remaining and newly arrived mRNA vaccines will be allocated first to other groups prioritised for mRNA vaccines, if any (none in this example). Subsequently, vaccines will be distributed to other age groups without prioritisation (indicated with *), specifically adults and the elderly in this example.

Our setup includes two types of vaccines corresponding to those available in Belgium during the initial vaccination campaign^51^: mRNA and vector-based vaccines^52^. The BNT162b2 vaccine by Pfizer-BioNTech and the mRNA-1273 by Moderna are grouped as mRNA vaccines. Analogously, AZD1222 by Oxford-AstraZeneca and Ad26COV2S by Janssen are both grouped as vector-based vaccines. Each simulation day, the reported supply of mRNA and vector-based vaccines—corresponding to the actual doses delivered in Belgium since January 1, 2021^53^—is allocated to a selected group of individuals. Weekly delivery quantities are extrapolated into daily vaccine uptakes assuming an uniform distribution over the days of a week (Fig. 3a).

We define a vaccination strategy as a quintuple of the different vaccine types, relative to the considered age groups: Children (0–4), Youngsters (5–18), Young Adults (19–25), Adults (26–64) and Elderly (65+). The quintuple remains fixed throughout one simulation. When vaccinating the population according to a vaccination strategy, we select unvaccinated individuals of the appropriate age groups. The number of vaccines per age group is specified based on the reported time-specific vaccine supply. This supply is proportionally distributed to the different age groups based on their respective sizes. Vaccines are allocated exclusively to the uptake cohort (i.e., the set of individuals willing to accept the vaccine, determined by the uptake parameter). Consequently, the prioritisation logic does not wait for the hesitant portion of an age group to be vaccinated. When all willing members of a prioritised age group have received their doses, any remaining supply is immediately reallocated to other age groups. This mechanism ensures that eligibility expands to the next priority group as soon as demand in the current group is exhausted. Furthermore, this aligns with the four-month simulation horizon (Jan–May 2021), a period historically characterised by strict sequential prioritisation due to supply scarcity, before broader overlapping eligibility phases were introduced later that year. As an example, we present the vaccine administration for one of the evaluated strategies in Fig. 3b.

In each simulation, we target a vaccine uptake. Vaccination uptake is organised by household, so we randomly select households from the STRIDE population, until the target uptake levels are met. We refer to this random households selection as the *uptake cohort*. During the simulation, vaccines will be allocated only to household members in the *uptake cohort*, considering the age restrictions outlined in the vaccination strategy.

For the vaccine to reach its full protection, it requires some time after its administration, as neutralising antibodies and virus-specific T cells must be produced^54^. We model this effect using a linear activation function, which linearly increases over a given time span, starting at the time when the first dose of the vaccine is administered. We assume the vaccine’s maximum efficacy is reached after 6 weeks, which mimics full vaccination scheme with a second dose after 4 weeks and maximum efficacy expected 2 weeks later^55^. For the Janssen vector-based vaccine, only one dose was administered. As these vector-based vaccines have a similar working mechanism, we assume the same activation function. We adopt differential vaccine efficacies for the Alpha VOC from the literature. For the mRNA vaccines, we assume a vaccine efficacy $VE_S = 95\%$ for the susceptibility, $VE_I = 95\%$ for infectiousness and $VE_D = 100\%$ for the propensity to protect from severe disease^40^. For the vector-based vaccines we assume $VE_S = 67\%$, $VE_I = 67\%$ and $VE_D = 100\%$^56^.

### Disease outbreak outcomes

There are two possible outcomes for an infectious disease outbreak: either the disease spreads beyond a local context to become a fully established epidemic or it fades out^57^. Therefore, the distribution of the epidemic sizes is bimodal, which is reflected by most stochastic epidemiological models^57^. In the context of this study, where we consider an ongoing COVID-19 epidemic, we can focus on the mode of the infection size distribution that is associated with the established epidemic. This distribution is known to be approximately Gaussian^9,58^. We note that this argument does not automatically hold for the hospitalisation size distribution, as for many infectious diseases, the likelihood to be hospitalised is not uniform within a population^59^. For COVID-19, hospitalisation rates rise exponentially with age^60^. Nonetheless, as for a particular scenario, we keep the contact reductions, uptake proportion and vaccine policy constant, we still expect a central trend that can be well approximated with a Gaussian.

To incorporate this prior knowledge in BFTS, we consider the reward distribution Gaussian with unknown mean and variance and assume an uninformative Jeffreys prior $(\sigma )^{-3}$ on $(\mu , \sigma ^2)$^61^. This prior leads to the non-standardised t-distributed posterior, that we truncate on the interval [0, 1] as we know the arm’s means are in this interval. The formal derivation for this posterior can be found in Appendix A.

### COVID-19 bandit

In the COVID-19 setting, we aim to find the vaccine allocation strategy that minimises the proportion of the population affected at the end of the simulation (i.e., the attack rate, abbreviated as AR). This rate can be estimated with regards to infections (ARI) or hospitalisations (ARH). To minimise the attack rate, we take the complement as a reward signal: $1-ARI$ for infections and $1-ARH$ for hospitalisations.

In operational terms, an arm in this setting corresponds to a single, unique vaccination strategy. As defined in the ‘Vaccine allocation’ section, a vaccination strategy is determined by assigning one of three options (mRNA vaccine, vector-based vaccine, or no priority) to each of the five age groups. For example, a single arm $a_{k}$ might represent the specific assignment quintuple Children: None, Youngsters: mRNA , Young adults: Vector-based , Adults: Vector-based , Elderly: mRNA). Pulling this arm triggers a STRIDE simulation where this specific allocation logic is applied. Since there are 3 options for each of the 5 age groups, the bandit explores a search space of $3^5$ distinct arms (i.e., 243 total strategies). In order not to waste any vaccines, we disregard all arms that do not use both types of vaccines, which results in a bandit with 180 arms.

While the bandit learns, it pulls an arm based on its sampling strategy. This arm is then translated to a corresponding vaccination strategy for each of the age groups. When pulling an arm, the bandit runs a STRIDE simulation for 4 calendar months, where the chosen vaccination strategy is executed until the end of the simulation.

A key feature of our setting is the distinction between the public health decision (i.e., the arm) and the environmental constraints. We model the vaccine supply, including scarcity, production ramp-up, and daily dosage limits, as an exogenous environmental factor, implemented in the epidemiological model. Consequently, the arms in our bandit framework represent the vaccine allocation strategy (i.e., the eligibility and prioritisation of age groups) rather than the resource volume itself. The bandit algorithm chooses an arm (i.e., a vaccine allocation strategy), and the environment returns a reward based on how well that policy performed given the current supply constraints. This ensures that trade-offs are not hard-coded assumptions, but are learned by the algorithm as it navigates the scarcity imposed by the environment.

## Results

In this section, we evaluate the performance of our multi-armed bandit framework and conduct a use case to investigate the public health impact of COVID-19 vaccine allocation, under distinct contact reduction schemes and vaccine uptake proportions. We begin by establishing a ground truth in a baseline scenario to validate the algorithm’s learning efficiency and accuracy in identifying the optimal vaccination strategies for each contact reduction scheme. Subsequently, we apply the framework to analyse COVID-19 vaccination policies under various contact reduction schemes. In this complex epidemiological setting, where exhaustive simulation is computationally prohibitive, we deploy the bandit framework to identify the top-performing strategies for minimising infections and hospitalisations. Through this framework, we analyse the resulting age-group prioritisations, social contact reductions, and the impact of varying vaccine uptake levels.

### Establishing a ground truth to evaluate the framework

During a pandemic, the efficient distribution of vaccines is crucial to reach the largest possible number of people. However, in practice, vaccine uptake is often lower than expected, e.g., due to factors such as vaccine hesitancy^43^. Furthermore, the European Centre for Disease Prevention and Control has emphasised the need for interventions to boost vaccine uptake to effectively control COVID-19^62^. In Belgium, a vaccine uptake rate of 75.7% was recorded later in 2021^63^, and accordingly, we adopt a vaccine uptake proportion of 75% in our simulations.

**Fig. 4:**
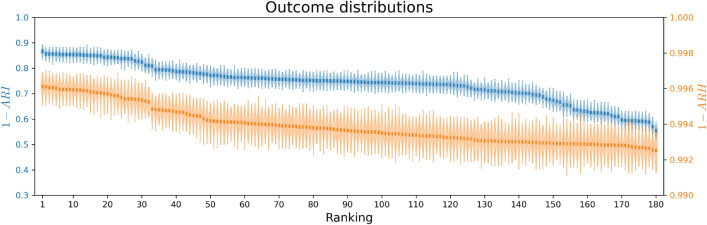
Ground truth of all vaccine allocation strategies for the baseline contact reduction scheme with 75% uptake, ranked based on 100 stochastic simulations. Ground truth of all vaccine allocation strategies, ranked based on infections (1 - ARI) and hospitalisations (1 - ARH). We note that the ranking based on infections versus hospitalisations does not necessarily match, here we show an independent ranking for each of the criteria.

To validate our method, we establish a *Baseline* scenario with a 75% uptake proportion (Table 1), where we obtain 100 simulation replicates for each of the vaccine allocation strategies, using the STRIDE stochastic individual-based model. This ground truth will be used to assess the performance of the algorithms to identify the true set of optimal strategies. Figure 4 shows the reward distributions for 100 simulation replicates of each of the vaccine allocation strategies (i.e., bandit arms).

**Fig. 5:**
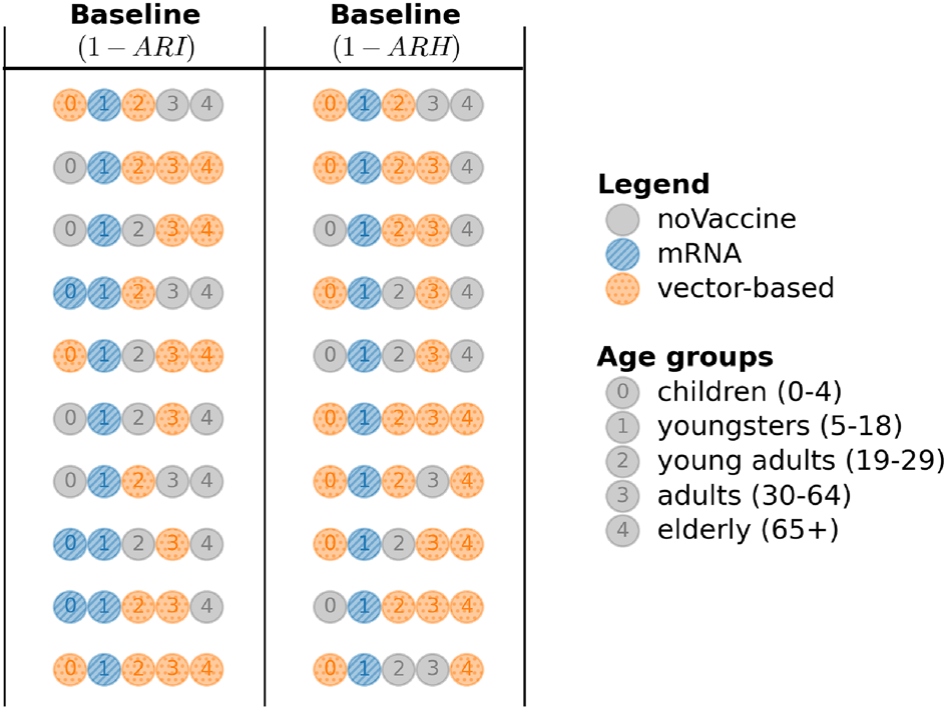
Ground truth of the top-10 vaccination strategies for the baseline scenario when minimising the infection (ARI) and hospitalisation (ARH) attack rates. Each strategy in the top-10 strategies is represented by 5 numbered circles, each representing a specific age group as highlighted in the legend. The colour of the circle indicates which vaccine type is being prioritised for the given strategy. For example, the first strategy when optimising for ARI prioritises vector-based vaccines for children and young adults. Youngsters receive priority for mRNA vaccines, while adults and elderly receive no vaccine priority.

The true *m*-top vaccination strategies demonstrate a distinct trend for the infection attack rate ARI and the hospitalisation attack rate ARH (Fig. 5). Most noticeably, the top-10 strategies prioritise vaccinating youngsters with mRNA vaccines. Children do not receive a particular recommendation in the top-10 strategies for ARI, as all vaccine types, including no vaccine, are present in these top-10 strategies. This indicates that assigning a particular vaccine type priority to children is less critical when reducing infections. When optimising for ARH, children are prioritised and receive vector-based vaccines in 7 of the top strategies. Young adults, adults and elderly receive vector-based vaccines if they are prioritised. Any remaining vaccines will be distributed among the remainder of the unvaccinated population. As a result, all age groups will eventually be vaccinated once the target age groups have been covered. We observe some overlap between the best performing strategies for ARI and ARH, which is expected since reducing overall infections also contributes to lowering hospitalisations.

Using this ground truth, we compare the performance of BFTS, AT-LUCB and Uniform sampling. Uniform sampling aims to pull each arm an equal number of times by pulling the least-sampled arm at each timestep. Consequently, uniform sampling recommends the empirical *m*-top arms. We report the algorithms’ performances using two statistics^26^. The first statistic is the proportion of correctly recommended arms at time *t*,

8 $$\begin{aligned} \frac{|J^{(t)} \cap J^{\text {True}}|}{m}. \end{aligned}$$

$J^{\text {True}}$ denotes the true set of optimal arms, which we know via our ground truth, and $J^{(t)}$ denotes the set of recommended arms at time *t*. The second statistic is the sum of the means of the *m*-top arms at time *t*,

9 $$\begin{aligned} \sum _{a \in J^{(t)}} \mu _a. \end{aligned}$$

**Fig. 6:**
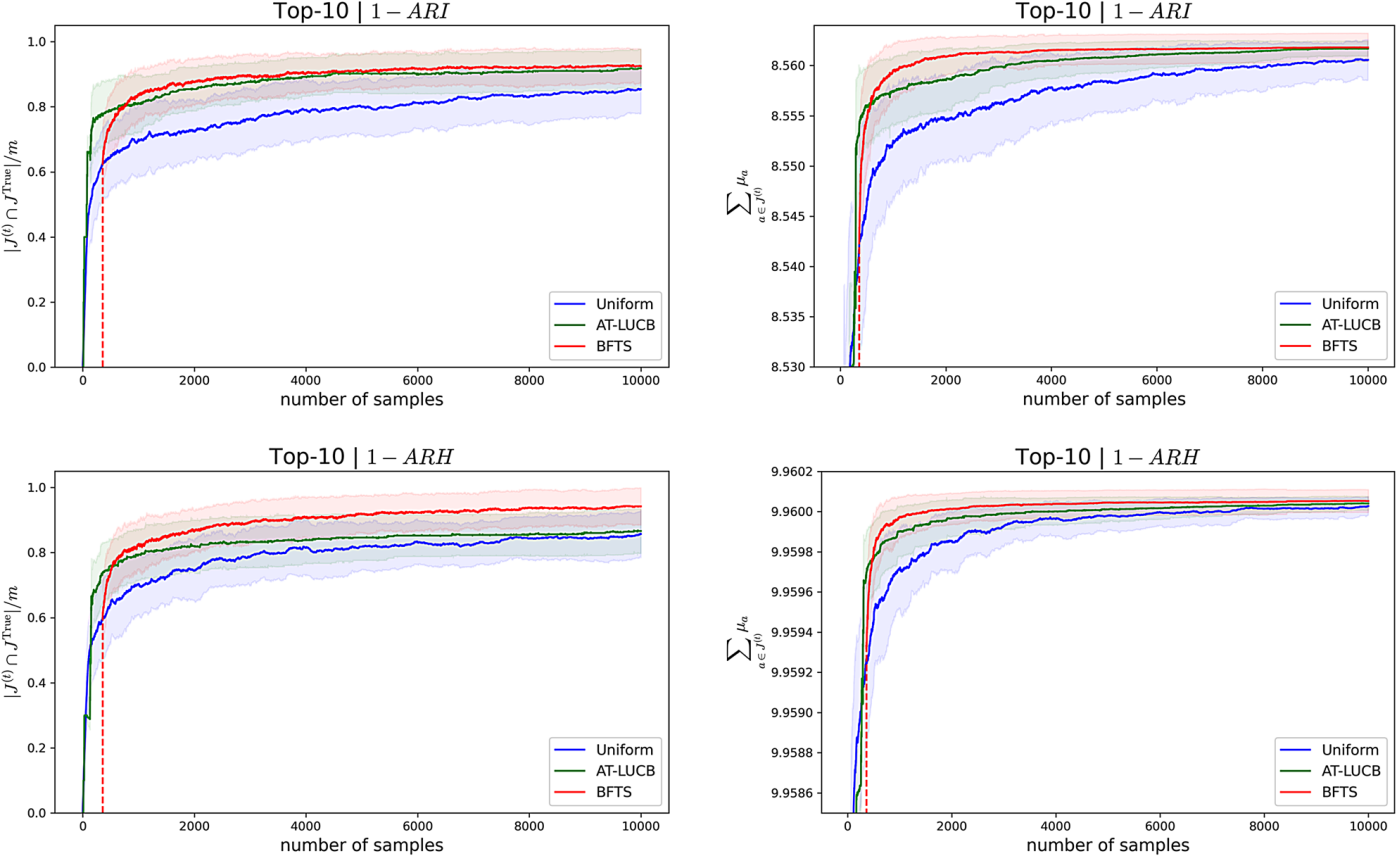
Learning curves for the ground truth based on infections (ARI) and hospitalisations (ARH), top vs bottom row, respectively. Left column: The average proportions of correctly ranked arms, with standard deviation. Right column: the average sum of true means, with standard deviation.

Note that uniform sampling and BFTS obtain one sample per time step, whereas AT-LUCB samples twice per timestep. We plot the results in terms of the number of samples (x-axis in Fig. 6) to facilitate a fair comparison. We consider truncated t-distribution posteriors for BFTS. Figure 6 shows the results of 100 simulation replicates per algorithm, over 10,000 samples, measured in terms of infections and hospitalisations. To obtain a proper posterior for BFTS, each arm’s posterior needs to be initialised twice^61^. In general, BFTS needs this short period to meet AT-LUCB’s performance, but quickly outperforms AT-LUCB after this warm-up period.

**Fig. 7:**
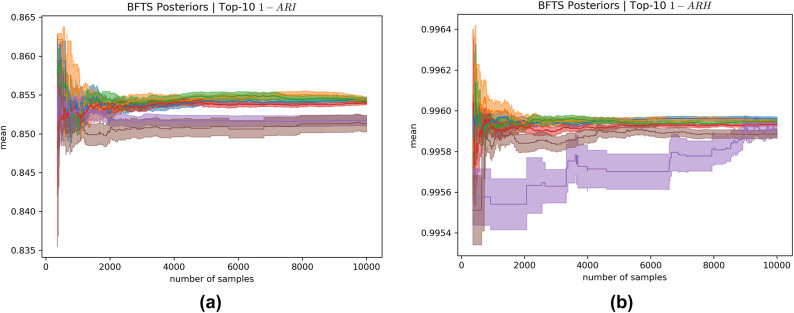
Posteriors for the *Baseline* scenario concerning (**a**) infections and (**b**) hospitalisations. The estimated means and uncertainties (standard deviations) are shown for the 3 arms above and the 3 arms below the decision boundary. Note that the arms closest to the decision boundary have a reduced uncertainty, as the bandit focused on these arms to reduce its uncertainty about the decision boundary.

Figure 7 shows the posteriors’ estimated means and uncertainty (standard deviation) for the 3 arms above and the 3 arms below the decision boundary of a single bandit run of BFTS. As BFTS pulls an arm, it reduces its uncertainty for that arm. However, as the true means are close to each other (see Fig. 4), there still remains uncertainty with regards to the estimated top-10 arms. We note that inspecting how these posteriors evolve over time presents an interesting way for decision makers to interpret and report the algorithm’s recommendations and the uncertainty associated with these recommendations.

### Analysing vaccination policies under various contact reduction schemes

We define a bandit with 180 vaccine allocation strategies, hence arms, to learn the top-10 vaccination strategies using BFTS, for the different contact reduction scenarios mentioned above (Table 1). Due to the computational burden of the STRIDE model accounting for the 11 million population for Belgium, running a single simulation, that is optimised and multi-threaded, takes approximately 5-6 minutes on the Genius and Hydra Vlaams Computer Centrum (https://www.vscentrum.be) high performance computing infrastructure, for our configurations. Each time the bandit pulls an arm, a new simulation is run. Consequently, the time required to perform experiments increases quickly due to the sequential nature of the bandit setting. As a result, we have set a limit of 2000 simulations (i.e., arm pulls) per experiment to obtain results equivalent to a uniform evaluation of 18,000 simulations. The 2000 simulations already correspond to about 1.5 weeks of computation on the Genius VSC high performance computing infrastructure. In the discussion section, we view further scaling of the simulations as a direction for future work.

For the *Baseline* scenario we obtained a ground truth to validate our results. For the other contact reduction schemes, we evaluate our bandit framework for what it was intended: to find the top strategies when evaluating each strategy independently is computationally unfeasible. Therefore, as we do not have a ground truth, we evaluate the quality of the obtained results by investigating the bandit’s decision uncertainty about the learned top vaccination strategies.

In this analysis, we investigate vaccine allocation strategies under distinct contact reduction schemes. The *Relaxed* scenario (Table 1) imposes $50\%$ contact reductions in the Workplace and Community, with Primary schools open at full capacity. Secondary schools operate at 50% capacity, while universities and colleges (i.e., Tertiary schools) are closed^13^. This scenario considers moderate restrictions at work and in the community, requiring a well-chosen vaccination strategy to counteract the additional contacts compared to the *Baseline* scenario. In the *Tertiary Education* scenario, we follow the same contact reductions as the baseline, with the exception of having Tertiary schools open at $70\%$ contact reductions. The *Secondary Schools* scenario explores the case where Primary and Secondary schools are open. As the *Baseline* scenario has shown that children should be prioritised when vaccinating, this scenario provides an interesting perspective as school contacts for children and youngsters are fully allowed. The *Relaxed Community* and *Relaxed Workplace* scenarios both consider a middle-ground between the *Baseline* and *Relaxed* scenario. The uncertainty analysis (based on the analysis of the posteriors) for all these scenarios can be found in Appendix D.

**Fig. 8:**
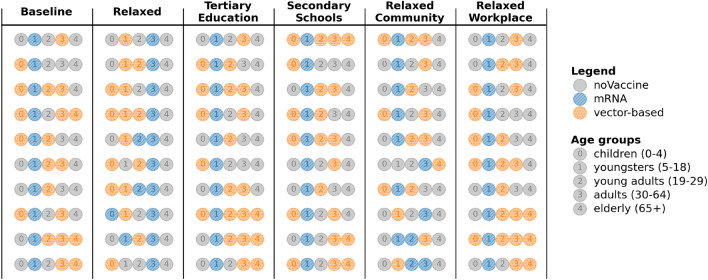
Learned top-10 vaccination strategies when minimising the infection attack rate (ARI) under various contact reduction schemes, under a 75% vaccine uptake proportion.

When minimising the infection attack rate (ARI), there is a clear trend regarding youngsters across the scenarios (Fig. 8). In all scenarios except the *Relaxed* and *Relaxed Community* scenarios, the top vaccination strategies exclusively prioritise mRNA vaccines for youngsters. In contrast, in the *Relaxed* scenario, our analysis recommends to prioritise adults with mRNA vaccines. As reducing infections is the priority, the most rewarding strategies are those that prioritise youngsters, young adults and adults as they are making more contacts compared to the *Baseline* scenario. It is worth noting that prioritisation of vaccines for the elderly seems linked to the social contact reduction scheme, suggesting that the social contacts made by the elderly may have a greater impact on the infection attack rate, in these scenarios.

**Fig. 9:**
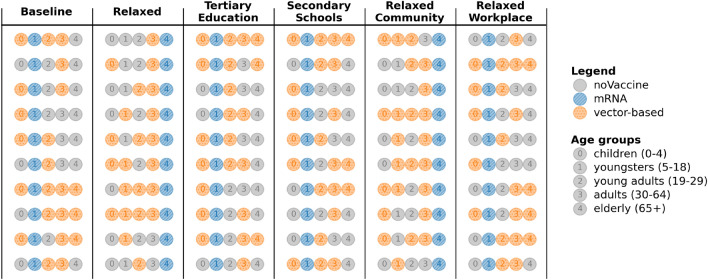
Learned top-10 vaccination strategies when minimising the hospitalisation attack rate (ARH) under various contact reduction schemes, under a 75% vaccine uptake proportion.

For the hospitalisation attack rate (ARH), we notice that youngsters are still prioritised with mRNA vaccines. However, there is a shift in focus for the *Relaxed* and *Relaxed Community* scenarios, where all top-10 strategies prioritise giving elderly mRNA vaccines (Fig. 9). Both scenarios allow more community contacts, where there is greater involvement of the elderly compared to school or work activities. As the older population is more likely to be hospitalised^60^, the bandit learns to vaccinate them first. Both vaccine types have the same efficacy in preventing severe disease and hospitalisations. However, mRNA vaccines are more effective in reducing susceptibility and infectiousness. Combined with their greater availability during the simulation, this makes them the preferred option for vaccinating and protecting the elderly from hospitalisation.

Interestingly, these results indicate for each contact reduction scenario which age group should be prioritised for vaccination. As the supply of mRNA vaccines is higher than the supply of vector-based vaccines throughout the experiments, choosing mRNA for an age group means that this age group will receive a majority of the vaccines, thereby reducing that particular age group’s impact on the attack rate. We refer back to Fig. 3 for an example of this prioritisation based on the supply. For example, in the *Baseline* scenario for ARH, even though hospitalisations were a priority, only three arms prioritise vaccinating the elderly as the impact of other age groups appears more important (Fig. 5). Similarly, the presence of multiple vaccine types for an age group in the top-10 strategies suggests that the specific vaccine type is less critical for that particular age group, as long as the individuals in this age group are vaccinated. For example, the *Baseline* scenario for ARI (Fig. 5) indicates less importance regarding the vaccination type when it comes to children, as mRNA and vector-based vaccines were both recommended options. We conclude that these results are heavily influenced by the effectiveness of the vaccines in significantly reducing transmission likelihood. With the emergence of new variants, these odds changed, which we reflect upon in the discussion section.

**Fig. 10:**
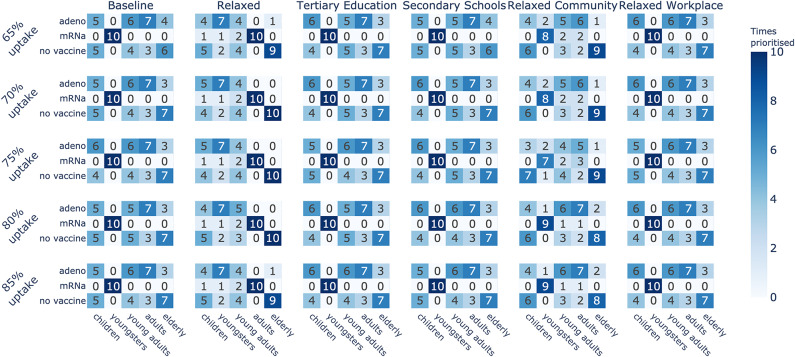
Priorities of vaccine types per age group across all uptakes, when optimising the infection attack rate (ARI) under various contact reduction schemes and uptake proportions.

**Fig. 11:**
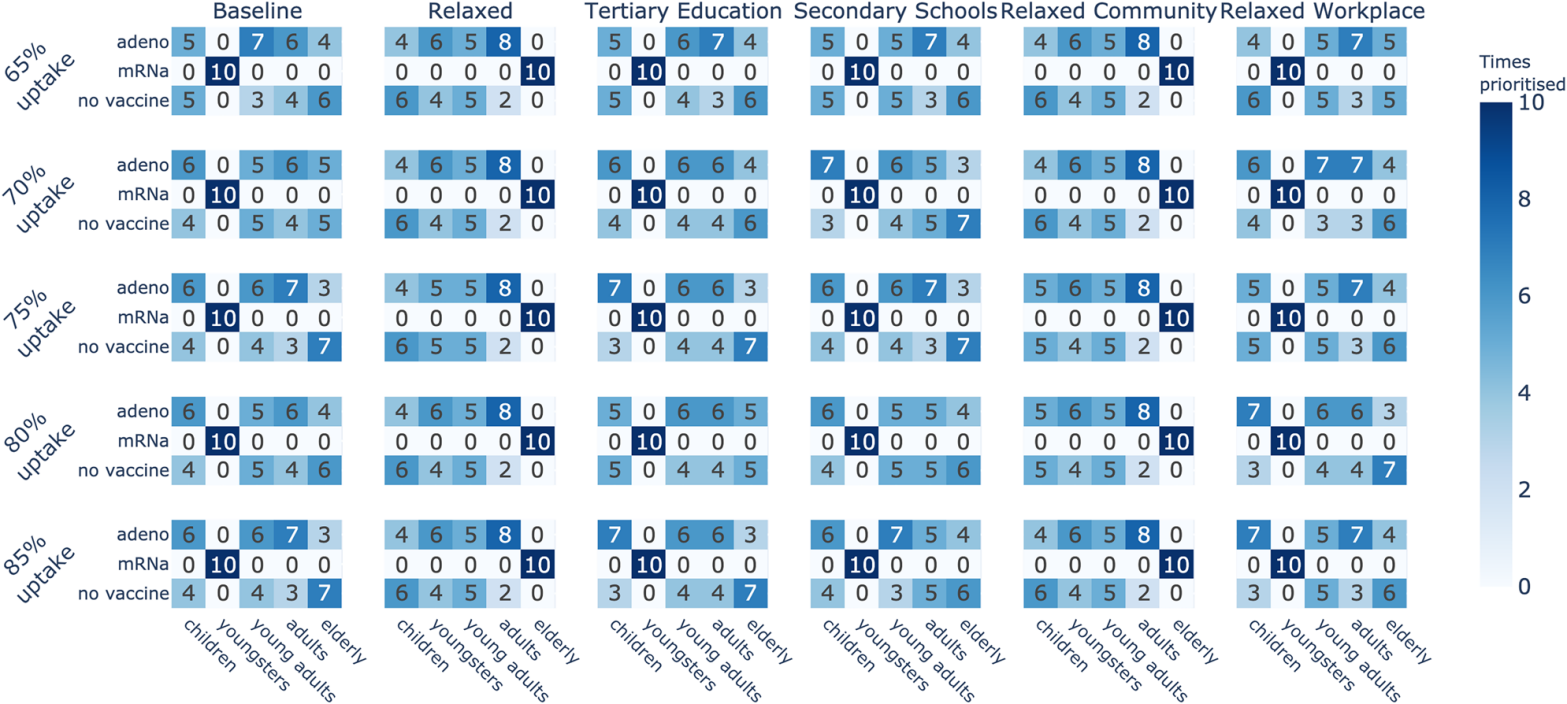
Priorities of vaccine types per age group across all uptakes, when optimising the hospitalisation attack rate (ARH) under various contact reduction schemes and uptake proportions.

To summarise these results, in Fig. 10 we show an overview of how often each age group is prioritised for a given vaccine type in the learned top-10 arms, under distinct vaccine uptake proportions. We note that across different uptake proportions, the number of times an age group is prioritised (for a given vaccine type) remains similar. While the overall age group priorities are similar for a given social contact reduction scheme, the best strategies learned might differ in their vaccine type combinations across age groups. Figure 11 shows an overview of the prioritisation when ARH is optimised. Here we observe similar priorities within a contact reduction scheme across the different uptake proportions. We present additional results on the specific top-10 strategies for all contact reduction schemes, for ARI and ARH, in Appendix E. Moreover, we show results for different uptake proportions of 65%, 70%, 80% and 85% in Appendix E.

## Discussion

In this work, we present a multi-armed bandit framework to study mitigation policies in individual-based epidemiological models. With this framework, we study vaccine allocation policies in a COVID-19 epidemic. Via a ground truth analysis, we show it is possible to efficiently learn the best strategies using a limited number of stochastic model evaluations. Additionally, the bandit allows policy makers to use the learned posteriors and their uncertainty to make informed decisions. Through our vaccine allocation study, we highlight the connection between targeted social contact reductions and the design of vaccine allocation policies.

Through our framework, we present a comprehensive retrospective analysis on the organisation of COVID-19 vaccination policies under different contact reduction schemes. Moreover, we investigate the impact of vaccine uptake proportions. Through our experiments, we show that the top vaccine allocation strategies follow a clear trend regarding the prioritised age groups and assigned vaccine type, which provides insights for future vaccination campaigns. When varying the overall uptake levels, our experiments suggest that this has limited influence on the optimal vaccine allocation policy design. Next to providing retrospective insights regarding COVID-19 vaccine allocations, we contribute a free software (GPL-licensed) framework that will facilitate the investigation of mitigation policies for future pandemics.

Our findings demonstrate the specific utility of using fine-grained IBMs for policy evaluation. We recover the general principle that targeting high-contact age groups (e.g., youngsters in the case of respiratory viruses) effectively reduces transmission, a finding consistent with compartmental modelling literature^44^. Beyond this established baseline, our analysis reveals critical nuances driven by the interaction of NPIs and vaccines, and assesses policy robustness under distinct vaccine uptake modalities. Specifically, we identify that the optimal strategy is conditional: in the Relaxed contact scenario, priority for infection reduction shifts towards adults and elderly. Furthermore, by modelling vaccine uptake at the household level, we provide insight into the robustness of these strategies against the clustering of unvaccinated individuals. Our analysis shows that the identified vaccine allocation priorities remain consistent across varying uptake levels (65%–85%), providing policymakers with confidence in the stability of such recommendations. Finally, the successful deployment of the anytime *m*-top bandit method demonstrates its utility as a generic framework for efficiently navigating the large search spaces inherent to realistic pandemic preparedness, allowing decision-makers to identify robust portfolios of strategies under uncertainty.

We make certain modelling assumptions. First, we keep contact reductions constant during a simulation. Second, we do not consider imported cases from abroad, motivated by the fact that we consider an ongoing epidemic that is mainly driven by intra-country contact dynamics. Third, our current policy structure relies on a static prioritisation ranking. While this mimics the strict eligibility phases seen during the four-month study period, campaigns conducted later in the progress of the pandemic might involve overlapping phases in later stages to maintain momentum^49^. For longer-term planning beyond this initial scarcity phase, a stateful reinforcement learning approach could be employed to learn dynamic thresholds for opening eligibility to new groups. Fourth, this study concerns a retrospective analysis that assumes that vaccine delivery dates are known. To reason about policies when the delivery scheme is uncertain, future work could extend the current framework to evaluate policy robustness against stochastic supply variations, identifying strategies that remain effective even when delivery schedules deviate from the projected timeline. Finally, regarding the implementation of NPIs, our model operationalises capacity limits (e.g., in schools or workplaces) as a proportional reduction in contact probabilities for all individuals in that setting, rather than explicitly simulating alternating attendance rosters (e.g., hybrid learning cohorts). While this effectively reduces the average transmission potential, it assumes a uniform reduction in mixing. In reality, physical capacity limits might create distinct, non-interacting subgroups, potentially leading to different local transmission dynamics (e.g., localised extinction within a cohort) that are homogenised within our mean-field approximation.

We note that the vaccine efficacies are representative for the start of the vaccination campaign, but as variants continued to emerge, vaccine efficacy regarding susceptibility and infectiousness has decreased significantly. Nonetheless, our study provides insights to optimal policies at the start of the vaccination campaign. We consider the evaluation of vaccination policies under the emergence of distinct VoC as future work. Additionally, we consider all age groups in the vaccine allocation study. However, as for the SARS-CoV-2 pandemic new vaccine platforms were trialed, these vaccines were only approved for 18 years and older at the start of vaccination campaign^64^. Our analyses do show that a rapid adoption of vaccines by children and/or youngsters could have an important impact on the epidemic and could allow lower contact reductions. This is an important consideration for future vaccination campaigns, which might target children earlier on, as the mRNA and vector-based vaccine platforms have now undergone rigorous evaluation^65,66^. In the context of COVID-19, it was shown that individuals might increase their contacts once they have been vaccinated^67^. We consider such behavioral aspects an interesting aspect to study in future work. Furthermore, as we consider a limited time period (4 months), vaccine waning is not considered in this study^68^. We do acknowledge that this would be interesting to consider for future work, when evaluating long term mitigation policies. Moreover, we consider it an interesting venue for future work to consider robustness of vaccination policies with respect to the emergence of variants.

In this work, we do not explicitly consider correlations between vaccine allocation strategies, to establish a generic policy evaluation framework that supports decision uncertainty. We note that the Bayesian exploration scheme will implicitly account for such correlations. While there are bandit algorithms that can exploit such correlations^69–71^, to the best of our knowledge there exist no Bayesian *m*-top exploration algorithms, which thus constitutes an interesting direction for future work.

Running sequential simulations for the bandit algorithm on STRIDE increases the time needed to conduct experiments. Therefore, our bandits framework would strongly benefit from parallelisation with regards to the pulled arms. We note that an extension of the Bayesian *m*-top algorithm with a delayed bandit approach constitutes an important venue for subsequent work^72^. Furthermore, additional optimisations and parallelisation to reduce the execution time of a single STRIDE simulation even more, could be explored.

While this study optimises infections and hospitalisations independently to identify the distinct extremes of the policy space, comparing these optima offers immediate practical guidance. Our results reveal a strategic dichotomy: strategies minimising infections consistently prioritise high-contact groups (e.g., youngsters) to build population-level immunity, whereas strategies minimising hospitalisations shift priority to the elderly, particularly when social contact restrictions are relaxed. The practical implication is that the optimal use of scarce vaccines is conditional. When NPIs are strict, targeting individuals active in contact generation (i.e., youngsters) is viable for both objectives. However, as society reopens (i.e., under relaxed contact reduction schemes), policy makers must pivot doses to the vulnerable (i.e., in the context of COVID-19, the elderly) to minimise severe outcomes. We emphasise that in this study, these specific trade-offs are identified within the context of the STRIDE model configuration and the specific characteristics of SARS-CoV-2. Consequently, caution is warranted when extrapolating these pivots to other pathogens with distinct transmission or severity profiles.

While our analysis reveals interesting insights in vaccine allocation strategies, it is important to note that these policies were learned within the limitations of the model used. To use the policies in a real epidemic emergency, a thorough validation is warranted.

For the vaccine allocation analyses under different contact reductions, we only allow the bandit a budget of 2000 samples, due to the computational burden of these analyses. On the one hand, this leaves room for uncertainty on the decision boundary, as was shown in our ground truth analysis. On the other hand, our ground truth analysis showed that BFTS is able to achieve good performance after 2000 steps, and our inspection of the posteriors of the contact reduction analyses confirmed this. We do stress that inspecting the posteriors is important, and when a high uncertainty is observed, this might warrant additional simulations. This is possible, using the anytime framework we present.

Our study assumes that household-based clustering of vaccine uptake serves as a reliable proxy for vaccine hesitancy. While supported by prior research^43^, this assumption warrants further scrutiny. Households capture collective decision-making tendencies, but it may overlook individual variability. Adolescents, for instance, often exhibit greater autonomy in health-related decisions compared to younger children^73,74^. Additionally, external influences such as peer pressure, workplace mandates, or targeted public health campaigns may shape individual attitudes beyond household-level norms^75^. To address these concerns, future work could involve accounting for demographic factors like age, education, and socioeconomic status that moderate decision-making within households. Moreover, alternative predictors such as geographic clustering or community-level factors could complement household-based analyses, offering a more nuanced understanding of vaccine hesitancy^76^.

We model social contact reductions as aggregate percentage decreases within broad contexts. Specifically, the Community maps to a diverse set of locations, including retail shops, hospitality venues (bars and restaurants), cultural institutions, leisure facilities, and public transport. Consequently, applying a uniform reduction (e.g., 70%) across this aggregate category limits our ability to evaluate targeted interventions, such as distinguishing between the closure of high-risk hospitality venues versus essential retail. While our current approach aligns with established methods^13^ and provides robust high-level insights, future work could integrate detailed venue-specific closure strategies.

In the COVID-19 vaccine allocation analysis, we modeled vaccine supply as an exogenous factor, reflecting the initial pandemic phase where production capacity is a hard constraint outside the policy maker’s control. Consequently, our vaccine allocation strategies focused strictly on eligibility and prioritisation. However, we acknowledge that in other public health contexts (e.g., distributing a strictly limited stockpile across different geographical regions) the allocation of the supply volume itself becomes a choice to be made by the policy maker. While studying such logistical settings would require a simulator adapted to regional dynamics, the multi-armed bandit framework remains directly applicable to learn the optimal policies. In such a case, the definition of one strategy (i.e., arm) would shift from eligibility rules to quantity distribution vectors, allowing the bandit algorithm to optimise logistical decisions.

In addition to the vaccine allocation policies studied here, our approach can be extended to explore other diseases and mitigation strategies for both disease and transmission, such as antiviral allocation strategies^77^. From an epidemiological perspective, future work may focus on the impact of universal testing approaches to mitigate the epidemic^14^, and repetitive testing in a school environment^78^. Similarly, the effect of superspreading^79^ and its impact on social distancing and vaccine allocation presents interesting venues for future research. Finally, a critical avenue for further inquiry is quantifying the trade-offs between maximising clinical outcomes and satisfying ethical constraints such as fairness. Pure utility maximisation (e.g., vaccinating spreaders) may conflict with equitable access for those at highest individual risk. Future work utilising multi-objective reinforcement learning could explicitly quantify the Pareto efficiency between infections, hospitalisations, and other objectives (e.g., fairness constraints), providing a comprehensive framework for the compromises discussed here^80,81^.

## Supplementary Information

Supplementary Information.

## Data Availability

The code for the bandit framework, the m-top algorithms and the COVID-19 experiments that were conducted in this paper is available at https://github.com/icimpean/m-top-covid. The vaccine extension to the STRIDE simulator is available at https://github.com/icimpean/stride/tree/vaccine.
