# Supplementary material for: Evaluating COVID-19 vaccine allocation policies using Bayesian m-top exploration
Source: Sci Rep. 2026 Apr 5;16:16365. doi: 10.1038/s41598-026-40787-x (PMC13212933; doi:10.1038/s41598-026-40787-x)
Supplement: Supplementary file 1 — Supplementary Information. [file 41598_2026_40787_MOESM1_ESM.pdf]

A Truncated t-distribution

We consider a Gaussian reward distribution with unknown variance and assume an uninformative Jeffreys prior $(\sigma)^{-3}$ on (μ, σ^2). Given rewards $\mathbf{r} = \{r_1, ..., r_n\}$, this prior leads to the non-standardised t-distributed posterior, that we truncate given that we know that the arms' means are in $[0, 1]$:

$$\mu \sim \mathscr{T}_{n,[0,1]}\left(\mu_0 = \frac{\sum_{i=1}^n r_i}{n}, \sigma_0^2 = \frac{\sum_{i=1}^n (r_i - \mu_0)^2}{n^2}\right). \tag{1}$$

Given the pdf $f(\cdot)$ of a non-standardised t-distribution $\mathscr{T}_\upsilon(\mu, \sigma^2)$

$$f(x) = \frac{\Gamma\left(\frac{\upsilon+1}{2}\right)}{\sigma\sqrt{\upsilon\pi}\Gamma\left(\frac{\upsilon}{2}\right)}\left(1 + \frac{(x-\mu)^2}{\upsilon\sigma^2}\right)^{-\frac{\upsilon+1}{2}}, \tag{2}$$

and cdf $F(\cdot)$, we can compute the mean of the truncated non-standardised t-distribution using this normalised definite integral:

$$\frac{\int_0^1 x f(x) dx}{F(\frac{1-\mu}{\sigma}) - F(\frac{0-\mu}{\sigma})} \tag{3}$$

From this, we can derive an analytic expression by first considering the numerator:

$$\begin{aligned}
&\int_0^1 x f(x) dx \\
&= \int_0^1 x \frac{\Gamma\left(\frac{\upsilon+1}{2}\right)}{\sigma\sqrt{\upsilon\pi}\Gamma\left(\frac{\upsilon}{2}\right)}\left(1 + \frac{(x-\mu)^2}{\upsilon\sigma^2}\right)^{-\frac{\upsilon+1}{2}} dx \\
&= \int_{x=0}^{x=1} \sigma \frac{x-\mu+\mu}{\sigma} \frac{\Gamma\left(\frac{\upsilon+1}{2}\right)}{\sqrt{\upsilon\pi}\Gamma\left(\frac{\upsilon}{2}\right)}\left(1 + \frac{(x-\mu)^2}{\upsilon\sigma^2}\right)^{-\frac{\upsilon+1}{2}} \frac{1}{\sigma} dx, \\
&\quad u = \frac{x-\mu}{\sigma}, du = \frac{1}{\sigma} dx \\
&= \int_{u=\frac{0-\mu}{\sigma}}^{u=\frac{1-\mu}{\sigma}} (\sigma u + \mu) \frac{\Gamma\left(\frac{\upsilon+1}{2}\right)}{\sqrt{\upsilon\pi}\Gamma\left(\frac{\upsilon}{2}\right)}\left(1 + \frac{u^2}{\upsilon}\right)^{-\frac{\upsilon+1}{2}} du \\
&= \int_{u=\frac{0-\mu}{\sigma}}^{u=\frac{1-\mu}{\sigma}} \sigma u f(u) du + \int_{u=\frac{0-\mu}{\sigma}}^{u=\frac{1-\mu}{\sigma}} \mu f(u) du
\end{aligned} \tag{4}$$

Substituting this in Equation 3, we have:

$$\begin{aligned}
&\frac{\int_{u=\frac{0-\mu}{\sigma}}^{u=\frac{1-\mu}{\sigma}} \sigma u f(u) du + \int_{u=\frac{0-\mu}{\sigma}}^{u=\frac{1-\mu}{\sigma}} \mu f(u) du}{F(\frac{1-\mu}{\sigma}) - F(\frac{0-\mu}{\sigma})} \\
&= \sigma \mathbb{E}\left[u \mid \frac{-\mu}{\sigma} \leq u \leq \frac{1-\mu}{\sigma}\right] + \mu
\end{aligned} \tag{5}$$

B Bayesian analysis of BFTS

This section performs a Bayesian analysis of BFTS[1], motivating its *pure exploration* strategy. We present two heuristics that form the basis of BFTS's exploration strategy, related to their probability of error.

In this Bayesian framework, we reason about the full distribution over bandits. Consequently, the actual means μ are unknown, and we assert our belief over μ given

$$\pi(\cdot \mid \mathscr{H}^{(t-1)}), \tag{6}$$

i.e., the prior belief over the means $\pi(.)$ conditioned on the observed history

$$\mathscr{H}^{(t-1)} = \left\{a^{(i)}, r^{(i)}\right\}_{i=1}^{(t-1)} \tag{7}$$

at time t.

We define $\Psi_\rho(\theta^{(t)})$ as the ρ ordered arm. We specify the random variables A_ρ^π as the ρ-ranked arms according to the prior belief, and A_ρ^{TS} as the ρ-ranked arm according to Thompson sampling (TS):

$$\begin{aligned} A_\rho^\pi &= \Psi_\rho(\mu) \\ A_\rho^{TS} &= \Psi_\rho(\theta^{(t)}) \end{aligned} \tag{8}$$

As TS is a *probability matching* algorithm[2,3], it samples directly from the belief asserted in Equation 6. Formally, this is defined as:

$$P(A_\rho^{TS} = \cdot \mid \mathscr{H}^{(t-1)}) = P(A_\rho^\pi = \cdot \mid \mathscr{H}^{(t-1)}) \tag{9}$$

We define $\rho^+ \in [1,\ldots,m]$ and $\rho^- \in [m+1,\ldots,K]$. Using this notation, we can express the true optimal arm set J^* and recommended arm set J^{TS} as:

$$\begin{aligned} J^* &= \{A_{\rho^+}^\pi \mid \forall \rho^+\} \\ J^{TS} &= \{A_{\rho^+}^{TS} \mid \forall \rho^+\}, \end{aligned} \tag{10}$$

we refer to $\overline{J^*}$ as the complement of J^*, i.e., the set of all arms excluding J^*. Note that both J^* and J^{TS} are random variables, as they are expressed as a union of random variables. We use $P_t(.)$ to denote a probability that is conditioned on the observed history $\mathscr{H}^{(t-1)}$ at time t:

$$P_t(.) = P(. \mid \mathscr{H}^{(t-1)}) \tag{11}$$

Given this framework, we identify two heuristics that underlie BFTS' sampling strategy.

Heuristic 1 *The expectation that BFTS wrongly ranks an arm that is believed to be optimal is bounded by the probability that BFTS wrongly ranks the arm on the sub-optimal side of the decision boundary:*

$$\mathop{\mathbb{E}}_{\rho^-}[P_t(A_{\rho^-}^{TS} \in J^*)] \leq P_t(A_{m+1}^{TS} \in J^*) \tag{12}$$

Given this inequality, we expect that sampling the m+1-th arm will reduce $\mathbb{E}_{\rho^-}[P_t(A_{\rho^-}^{TS} \in J^*)]$.

Heuristic 2 *The expectation that BFTS wrongly ranks an arm that is believed to be sub-optimal is bounded by the probability that BFTS wrongly ranks the arm ranked on the optimal side of the decision boundary.*

$$\mathop{\mathbb{E}}_{\rho^+}[P_t(A_{\rho^+}^{TS} \in \overline{J^*})] \leq P_t(A_m^{TS} \in \overline{J^*}) \tag{13}$$

Given this inequality, we expect that sampling the m-th arm will reduce $\mathbb{E}_{\rho^+}[P_t(A_{\rho^+}^{TS} \in \overline{J^*})]$.

These heuristics come from the fact that it is counterintuitive for TS to often order an arm as optimal when it is *believed* to be suboptimal. However, due to the stochastic nature of both the bandit and TS, it is possible to end up with a posterior for which the heuristics do not hold. Notwithstanding, we argue that given the intuition behind probability matching, such events become unlikely when reasonable priors are chosen and BFTS' exploration strategy is followed.

We now show how the expectations in the heuristics relate to the probability of error. As such, given the heuristics, we can bound the probability of error with respect to both sides of the decision boundary (i.e., A_{m+1}^{TS} and A_m^{TS}), demonstrating that BFTS' exploration strategy is well-grounded.

First, we derive the bound in terms of A^{TS}_{m+1}:

$$
\begin{aligned}
P_t\left(J^* \neq J^{TS}\right) &= P_t\left(\bigvee_{\rho^-} A^{TS}_{\rho^-} \in J^*\right) \\
&\leq \sum_{\rho^-} P_t\left(A^{TS}_{\rho^-} \in J^*\right) \\
&= \frac{\sum_{\rho^-} P_t\left(A^{TS}_{\rho^-} \in J^*\right) \cdot (K-m)}{(K-m)} \\
&= \mathop{\mathbb{E}}_{\rho^-}\left[P_t\left(A^{TS}_{\rho^-} \in J^*\right)\right] \cdot (K-m) \\
&\overset{(H1)}{\leq} P_t\left(A^{TS}_{m+1} \in J^*\right) \cdot (K-m)
\end{aligned}
\tag{14}
$$

In the first step, we express the probability of error in terms of the arms that are ranked as sub-optimal by TS. In the second step, we apply a union bound. In the third and fourth step, we transform the sum to an expected value. In the final step, we apply Heuristic 1 ($H1$).

Following analogous arguments, we derive the bound in terms of A^{TS}_{m} by applying Heuristic 2 (full derivation in Supplementary Information):

$$
P_t\left(J^* \neq J^{TS}\right) \leq P_t\left(A^{TS}_{m} \in \overline{J^*}\right) \cdot m \tag{15}
$$

These insights motivate a uniform selection of the two arms on both sides of the decision boundary, as is reflected in BFTS (see Algorithm 2, lines 2 and 3 in the for loop).

The BFTS algorithm is constructed such that its sampling strategy is completely independent of its recommendation strategy. Likewise, in this analysis, we consider the belief that BFTS maintains over the problem, in terms of the random variable J^{TS} (Equation 10), rather than the statistic that is used to make recommendations (e.g., the mean of the posterior in our experiments). This observation shows that our analysis is independent from the statistic used to make recommendations with BFTS.

When inspecting other algorithms for the m-top setting, we observe that the decision boundary between the mth and $m+1^{\text{th}}$ arms also plays an important role. For example, the frequentist algorithm AT-LUCB samples two arms each step; the one with the smallest lower-bound among the m-top arms, and the one with the greatest upper-bound among the rest. This is analogous to choosing the optimal and sub-optimal arms that are closest to the decision boundary.

C STRIDE contact tracing configuration

Based on configurations by Willem et al.[4], for contact tracing we assume a 0.7 detection probability with a daily case finding capacity of 10.000 individuals. Tests have a 0.1 probability to be false negative results. There is a 1 day delay for detecting symptomatic individuals and 2 days delay for isolating infected individuals.

D COVID-19 experiments

D.1 65% uptake proportion

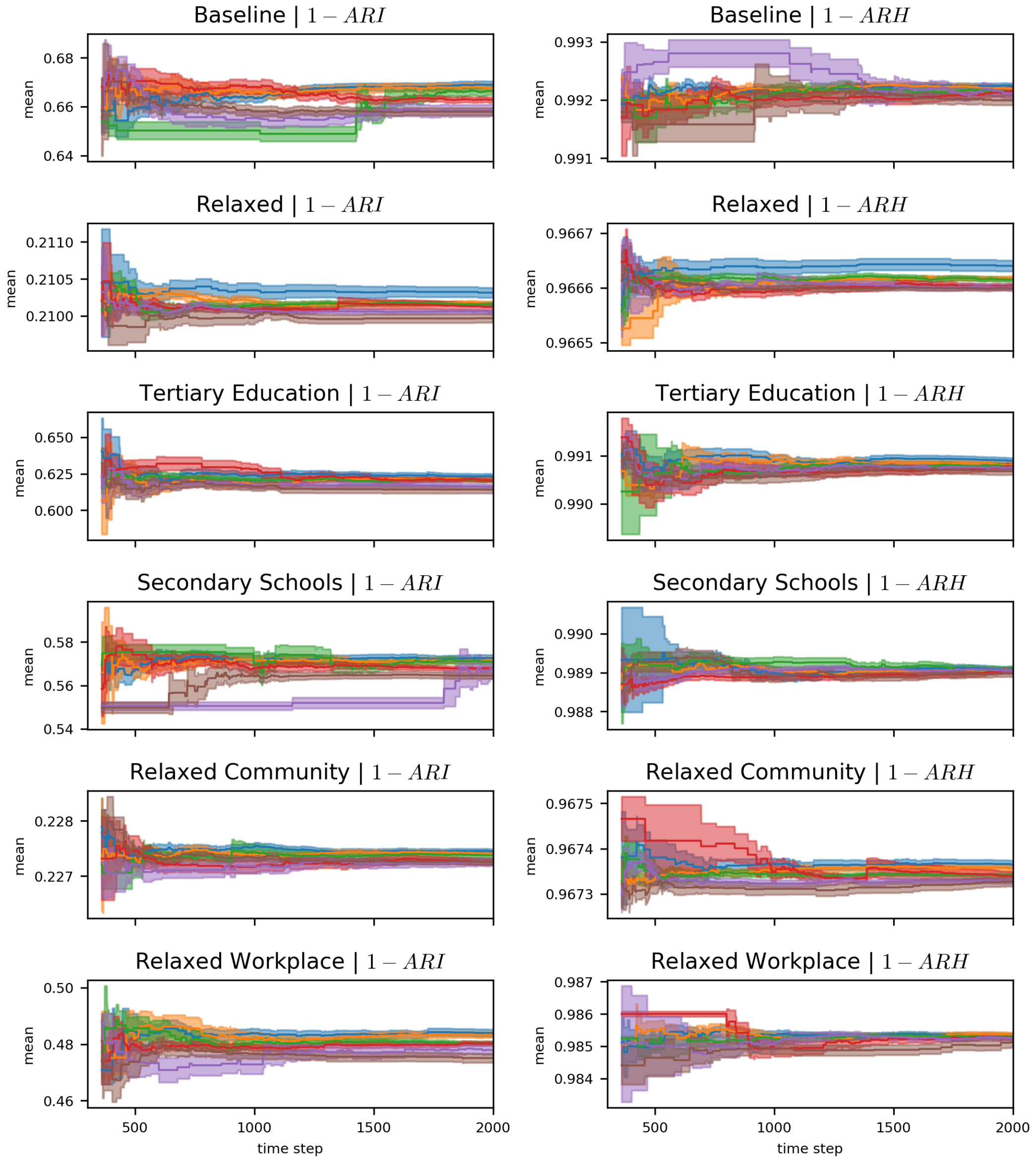

Figure 1. Estimated means and uncertainty (standard deviation) for the 6 arms around the decision boundary. (left) infections ARI. (right) hospitalisations ARH. For the top-10 strategies, these are the 8th-13th ranked arms under different contact reduction schemes, for a 65% vaccine uptake proportion.

D.2 70% uptake proportion

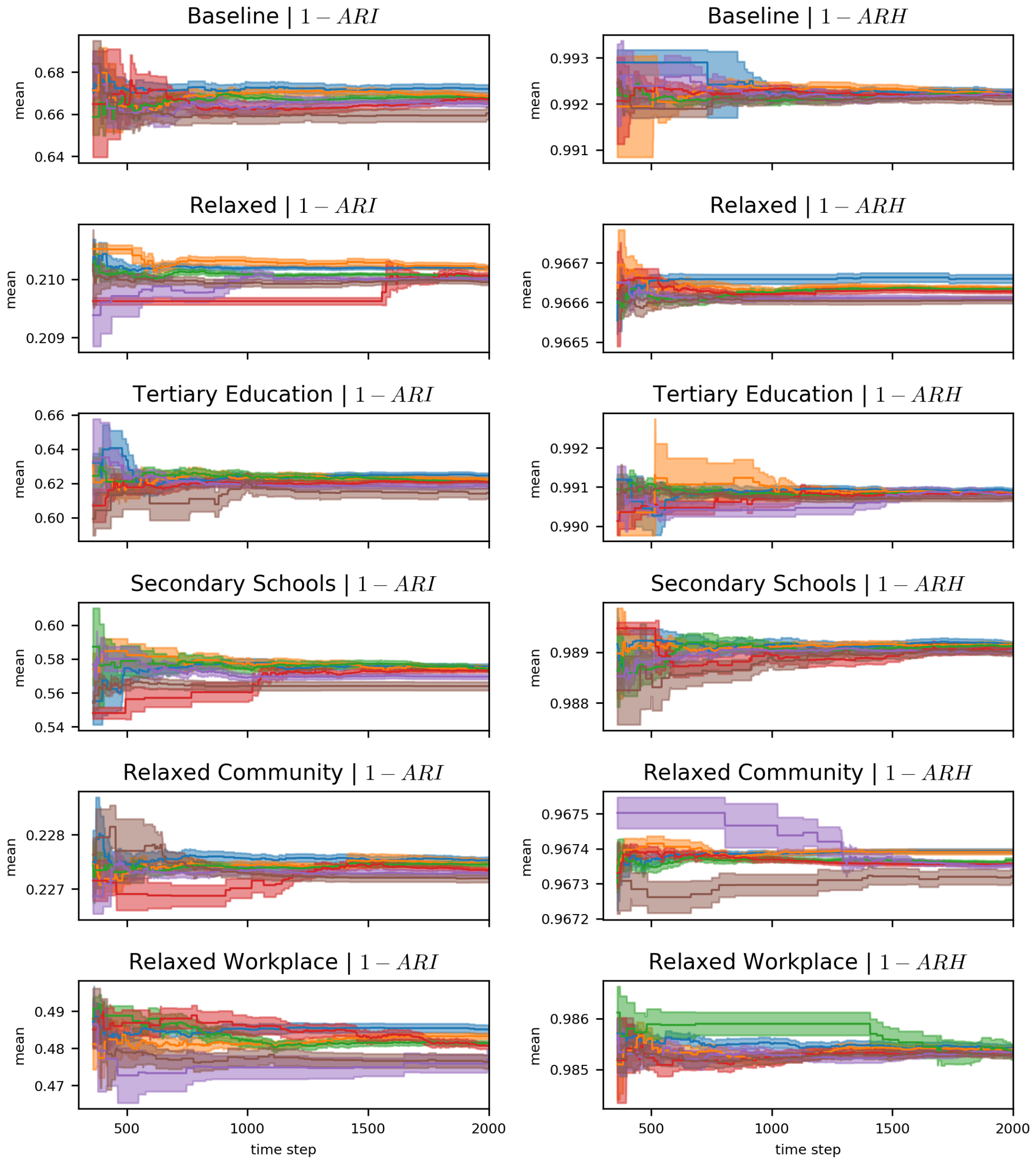

Figure 2. Estimated means and uncertainty (standard deviation) for the 6 arms around the decision boundary. (left) ARI. (right) ARH. For the top-10 top strategies, these are the 8th-13th ranked arms under different contact reduction schemes, for a 70% vaccine uptake proportion.

D.3 75% uptake proportion

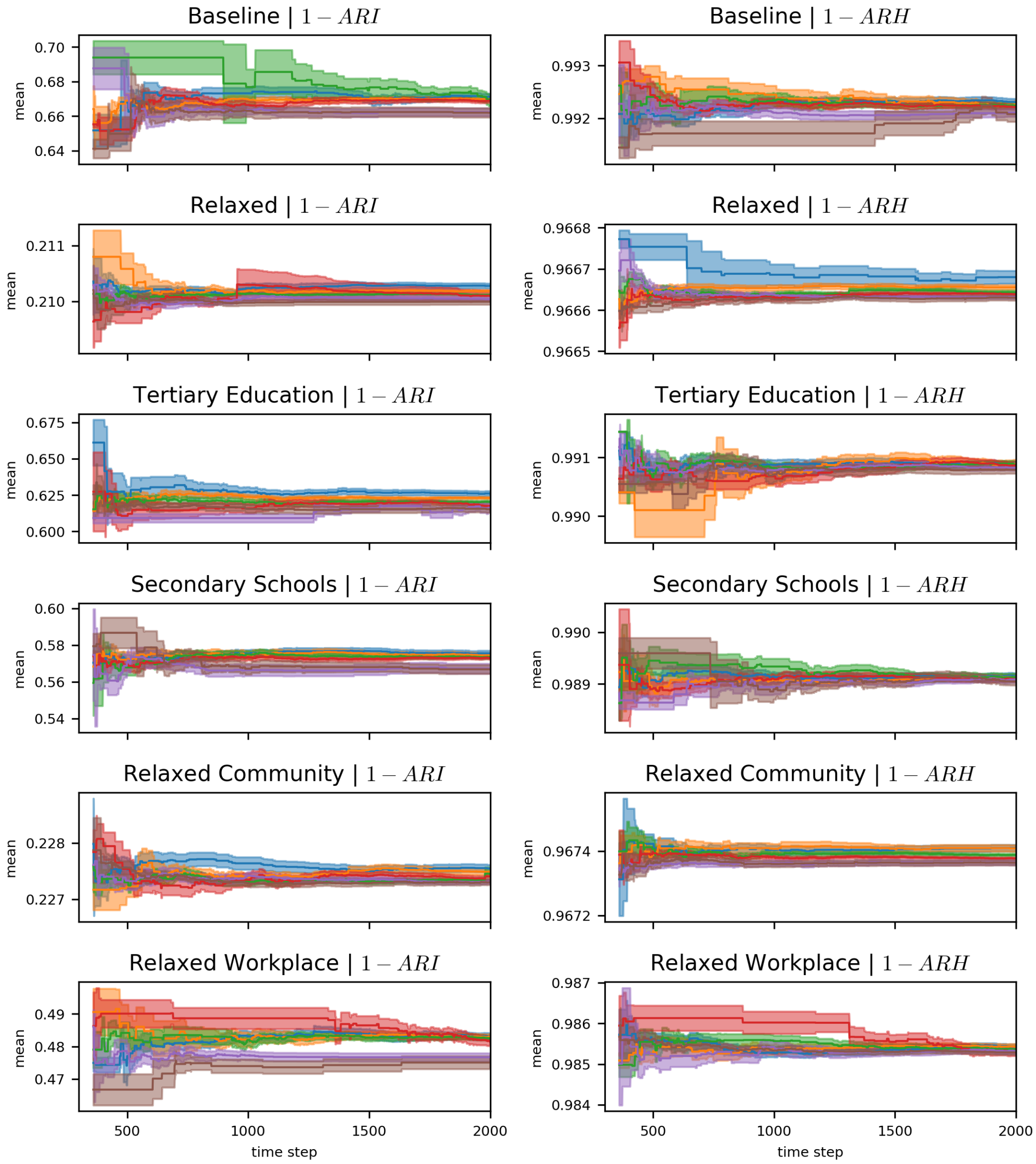

Figure 3. Estimated means and uncertainty (standard deviation) for the 6 arms around the decision boundary. (left) ARI. (right) ARH. For the top-10 strategies, these are the 8th-13th ranked arms under different contact reduction schemes, for a 75% vaccine uptake proportion.

D.4 80% uptake proportion

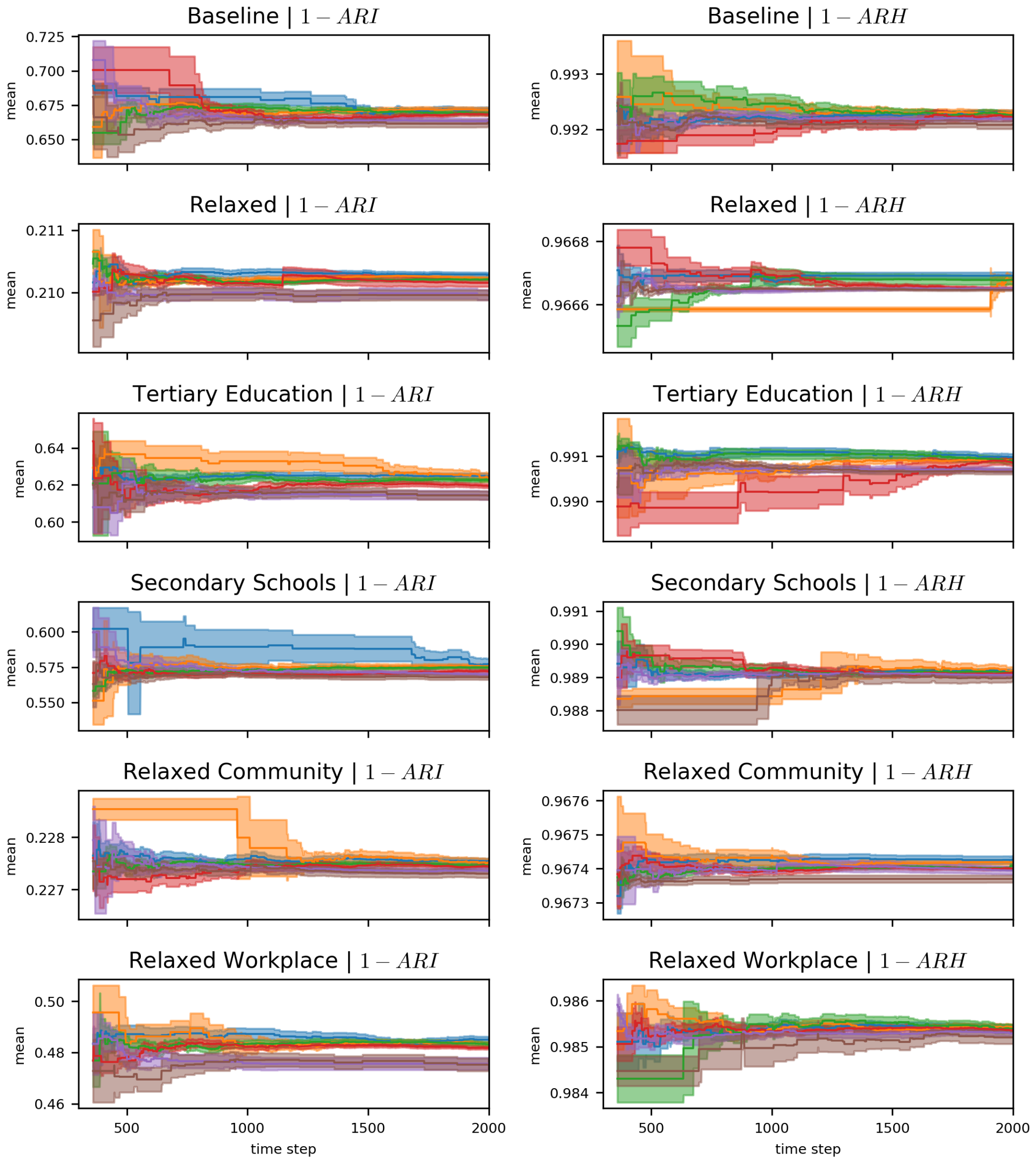

Figure 4. Estimated means and uncertainty (standard deviation) for the 6 arms around the decision boundary. (left) ARI. (right) ARH. For the top-10 strategies, these are the 8th-13th ranked arms under different contact reduction schemes, for a 80% vaccine uptake proportion.

D.5 85% uptake proportion

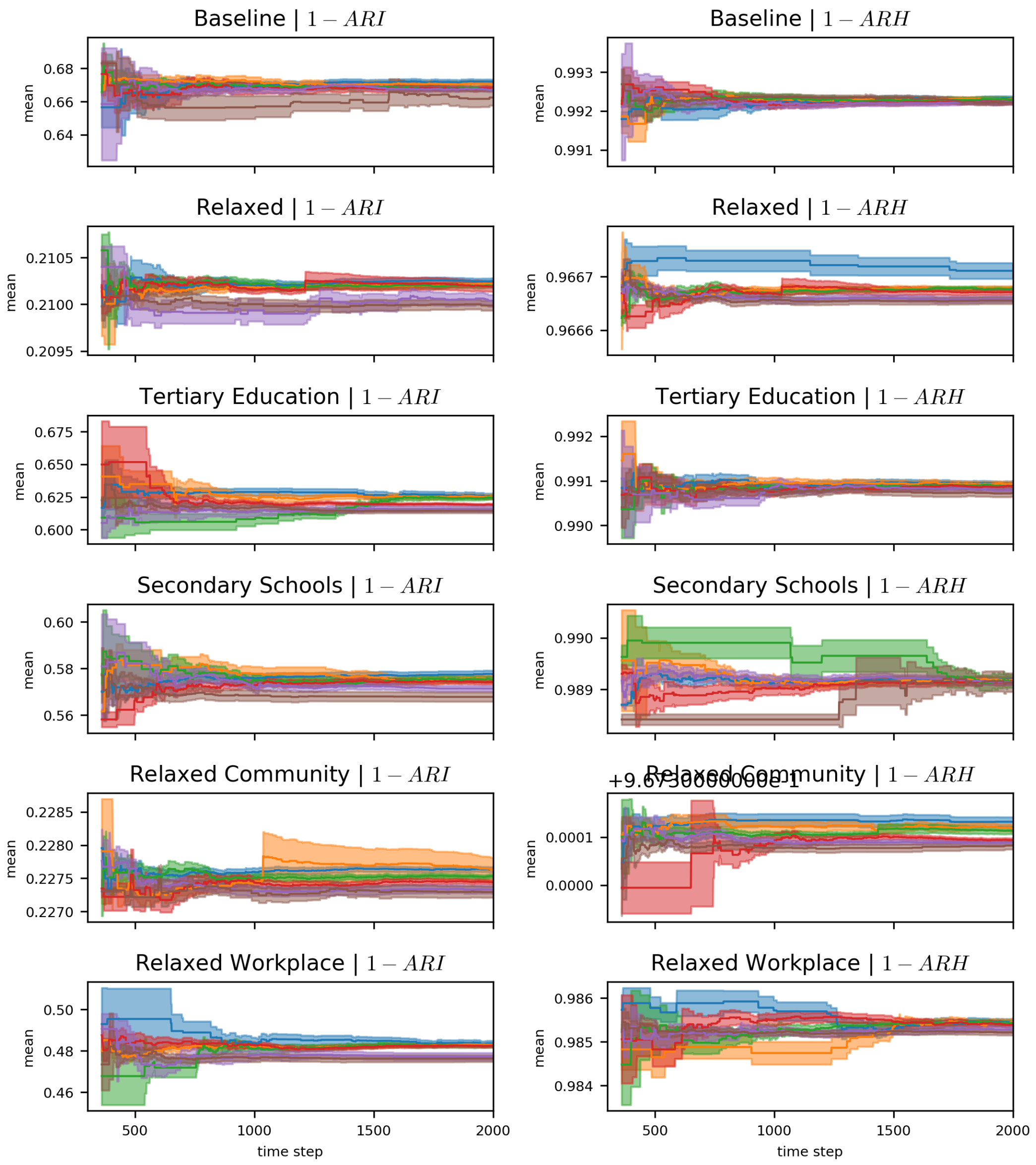

Figure 5. Estimated means and uncertainty (standard deviation) for the 6 arms around the decision boundary. (left) ARI. (right) ARH. For the top-10 strategies, these are the 8th-13th ranked arms under different contact reduction schemes, for a 85% vaccine uptake proportion.

E Top-10 vaccination strategies under different uptake percentages

E.1 65% uptake proportion

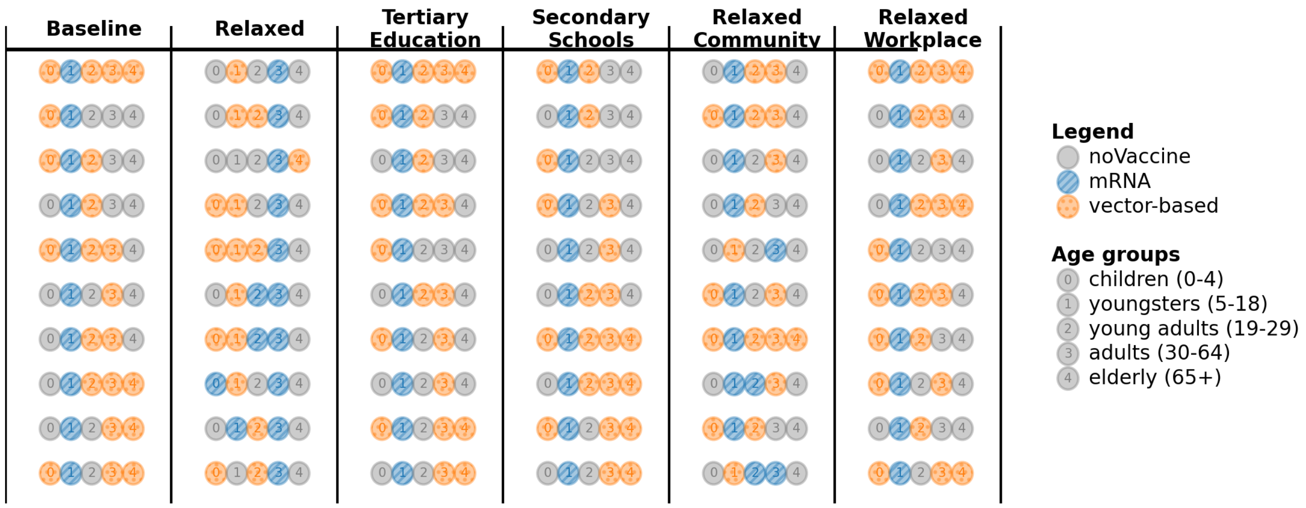

Figure 6. Learned top-10 vaccination strategies when minimising the infection attack rate (ARI) under various contact reduction schemes, under a 65% vaccine uptake proportion.

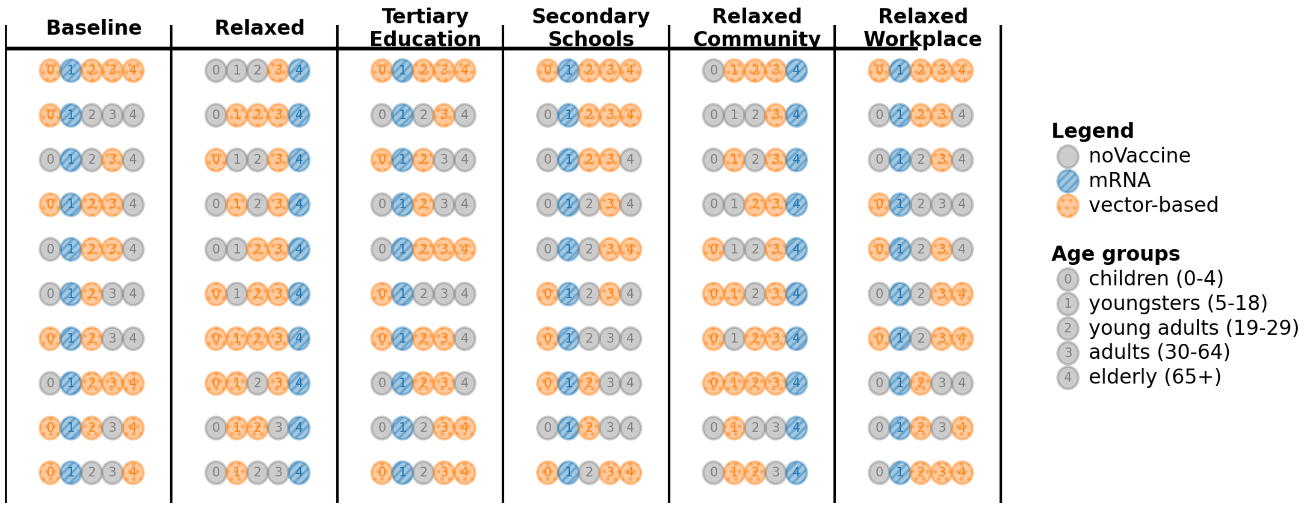

Figure 7. Learned top-10 vaccination strategies when minimising the hospitalisation attack rate (ARH) under various contact reduction schemes, under a 65% vaccine uptake proportion.

E.2 70% uptake proportion

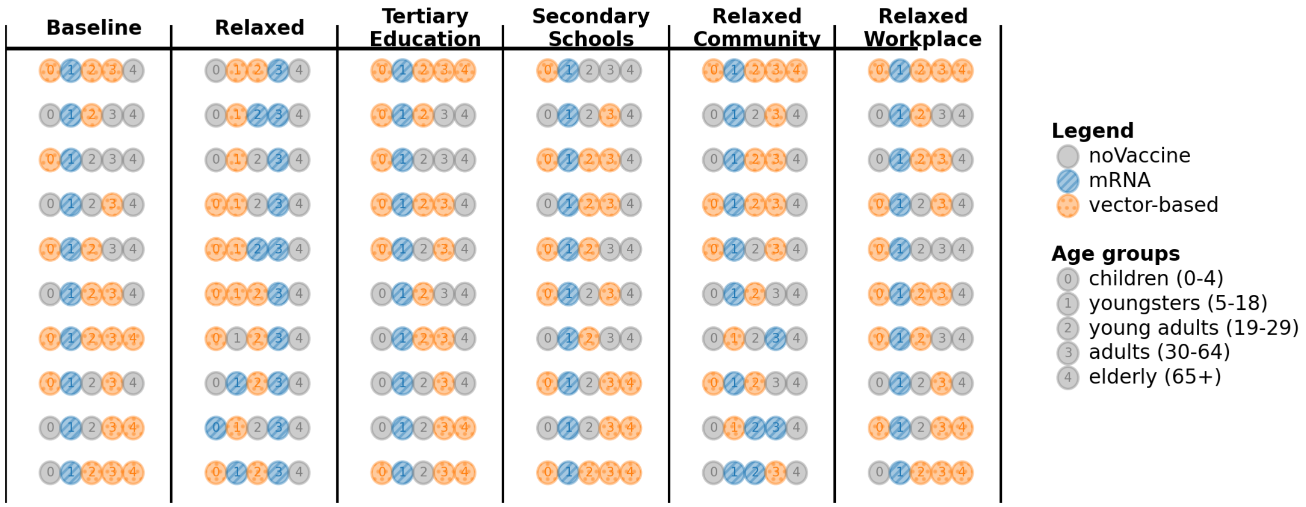

Figure 8. Learned top-10 vaccination strategies when minimising the infection attack rate (ARI) under various contact reduction schemes, under a 70% vaccine uptake proportion.

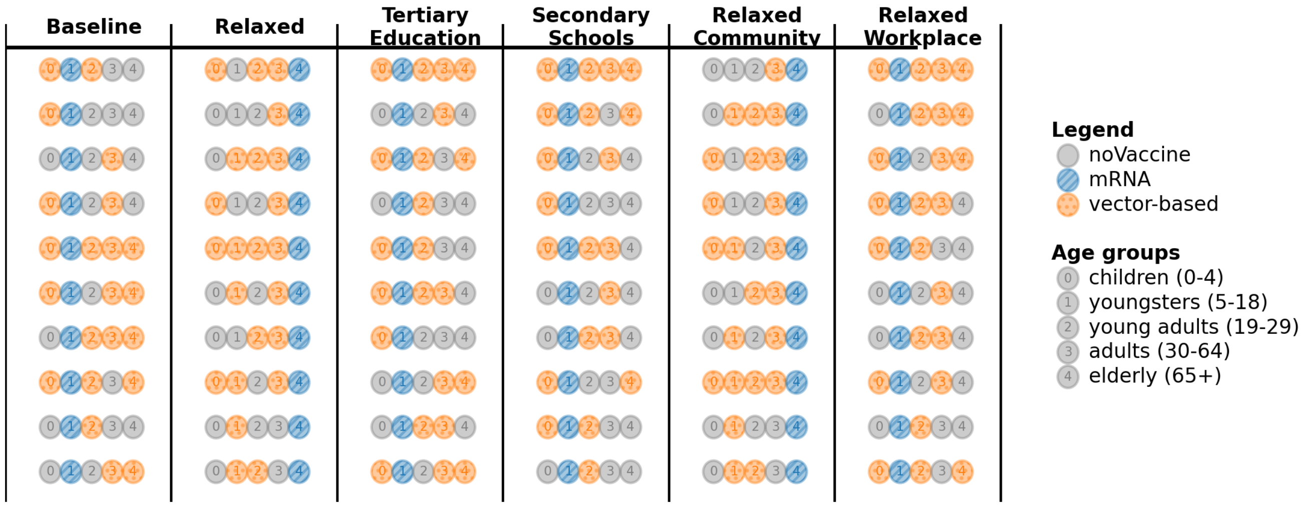

Figure 9. Learned top-10 vaccination strategies when minimising the hospitalisation attack rate (ARH) under various contact reduction schemes, under a 70% vaccine uptake proportion.

E.3 80% uptake proportion

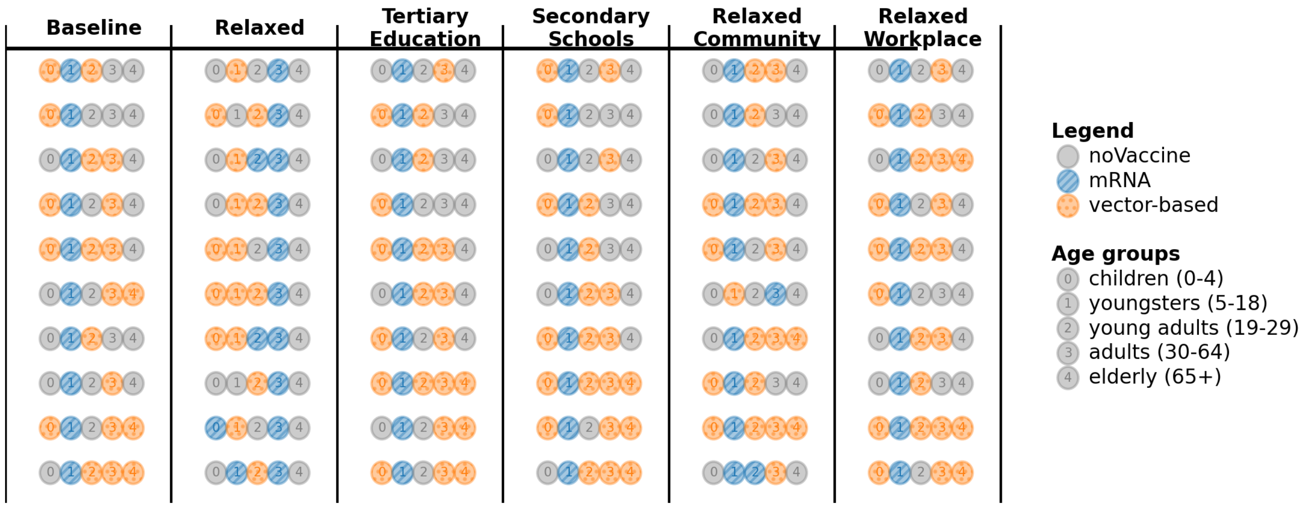

Figure 10. Learned top-10 vaccination strategies when minimising the infection attack rate (ARI) under various contact reduction schemes, under a 80% vaccine uptake proportion.

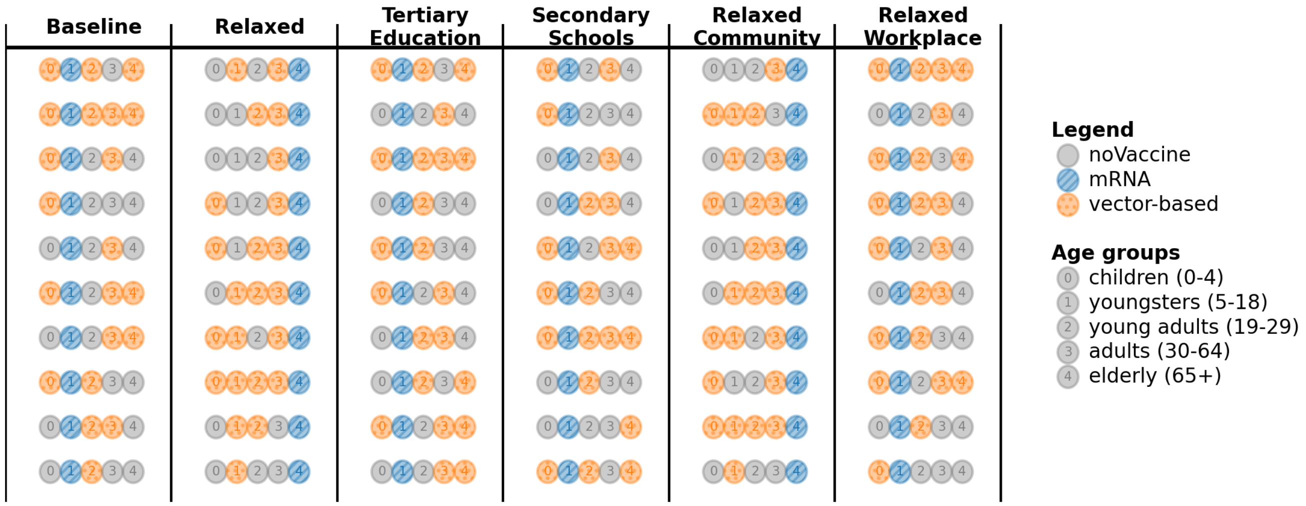

Figure 11. Learned top-10 vaccination strategies when minimising the hospitalisation attack rate (ARH) under various contact reduction schemes, under a 80% vaccine uptake proportion.

E.4 85% uptake proportion

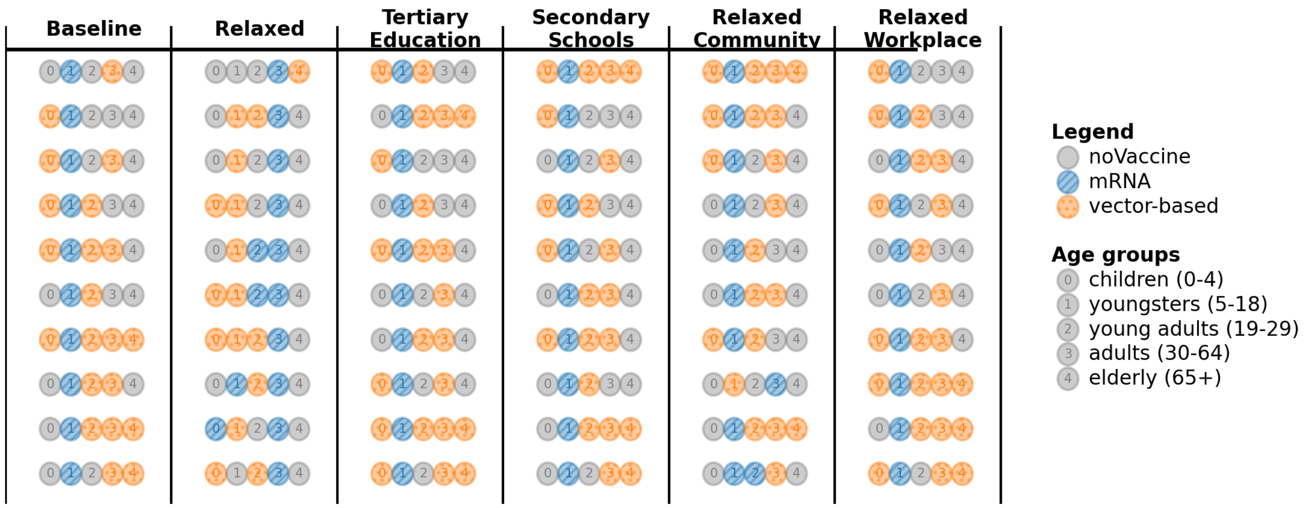

Figure 12. Learned top-10 vaccination strategies when minimising the infection attack rate (ARI) under various contact reduction schemes, under a 85% vaccine uptake proportion.

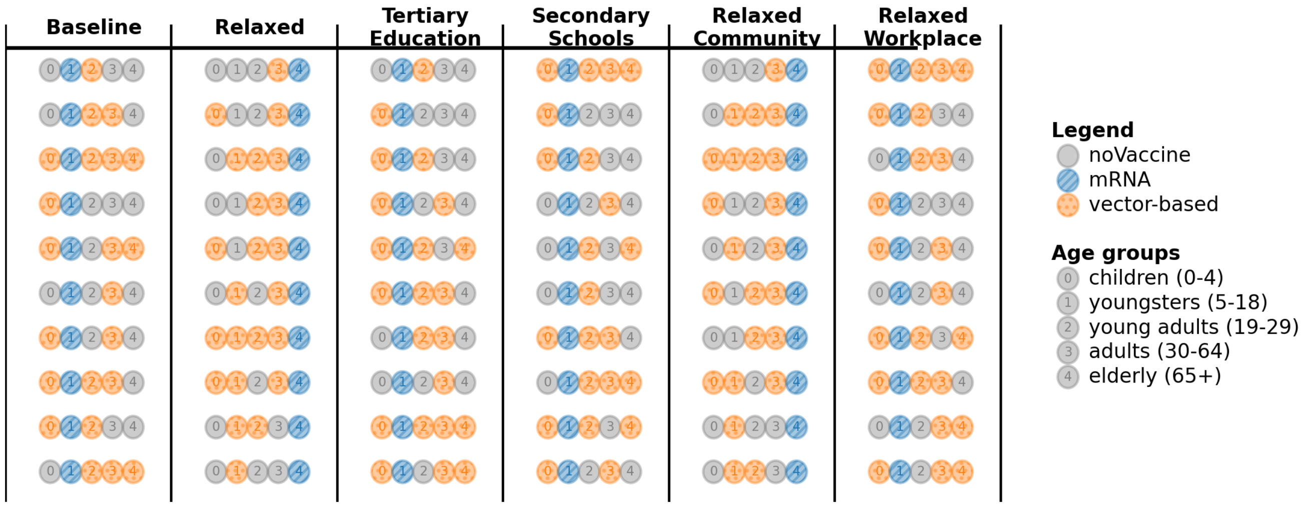

Figure 13. Learned top-10 vaccination strategies when minimising the hospitalisation attack rate (ARH) under various contact reduction schemes, under a 85% vaccine uptake proportion.

References

1. Libin, P. *et al.* Bayesian anytime m-top exploration. In *International Conference on Tools with Artificial Intelligence*, 1422–1428 (2019).
2. Agrawal, S. & Goyal, N. Analysis of thompson sampling for the multi-armed bandit problem. In *Conference on Learning Theory*, 39–1 (2012).
3. Russo, D. & Van Roy, B. An information-theoretic analysis of thompson sampling. *The J. Mach. Learn. Res.* **17**, 2442–2471 (2016).
4. Willem, L. *et al.* The impact of contact tracing and household bubbles on deconfinement strategies for COVID-19. *Nat. Commun.* **12**, 1–9 (2021).
